# A tri-modal contrastive learning framework for protein representation learning

**DOI:** 10.1016/j.crmeth.2026.101407

**Published:** 2026-04-15

**Authors:** Li Zhang, Han Guo, Leah Schaffer, Young Su Ko, Digvijay Singh, Hamid Rahmani, Danielle Grotjahn, Elizabeth Villa, Michael Gilson, Wei Wang, Trey Ideker, Eric Xing, Pengtao Xie

**Affiliations:** 1 Department of Electrical and Computer Engineering, University of California, San Diego (UC San Diego), La Jolla, CA 92093, USA; 2 Department of Medicine, UC San Diego, La Jolla, CA 92093, USA; 3 Department of Chemistry and Biochemistry, UC San Diego, La Jolla, CA 92093, USA; 4 School of Biological Sciences, UC San Diego, La Jolla, CA 92093, USA; 5 Department of Integrative Structural and Computational Biology, The Scripps Research Institute, La Jolla, CA 92037, USA; 6 Howard Hughes Medical Institute, UC San Diego, La Jolla, CA 92093, USA; 7 Skaggs School of Pharmacy and Pharmaceutical Sciences, UC San Diego, La Jolla, CA 92093, USA; 8 Department of Cellular and Molecular Medicine, UC San Diego, La Jolla, CA 92093, USA; 9 Department of Bioengineering, UC San Diego, La Jolla, CA 92093, USA; 10 Machine Learning Department, Carnegie Mellon University, Pittsburgh, PA 15213, USA; 11 Mohamed bin Zayed University of Artificial Intelligence, Masdar City, Abu Dhabi, UAE

**Keywords:** protein foundation model, multimodal learning, protein function prediction, protein property prediction

## Abstract

Protein foundation models, particularly protein language models, have shown strong success in learning meaningful protein representations using transformer architectures pretrained on large-scale datasets through self-supervised learning. These representations have proven effective for downstream tasks such as predicting protein functions and properties. However, most existing models focus solely on amino acid sequences, overlooking other informative modalities such as 3D structures and literature text. While some recent efforts incorporate multiple modalities, they often suffer from limitations in modality coverage or training strategy. To address this gap, we propose a multimodal pretraining framework that integrates three complementary modalities—protein sequences, structures, and literature text. Our method uses the sequence modality as an anchor and aligns the other two modalities to it via contrastive learning, enabling the model to capture richer and more holistic protein representations. Across a diverse set of downstream tasks, ProteinAligner outperforms state-of-the-art foundation models in predicting protein functions and properties.

## Highlights

• ProteinAligner aligns sequence, structure, and text via contrastive learning
• Encoders pretrained with multimodal alignment excel across protein prediction tasks
• ProteinAligner consistently surpasses state-of-the-art protein foundation models

## Motivation

Current protein language models (PLMs) primarily focus on amino acid sequences, often overlooking the critical biological insights contained in 3D structures and scientific literature. While structures provide essential spatial information for understanding folding and interactions, literature captures context-specific experimental insights. Existing multimodal efforts suffer from limited modality coverage, quantization noise in structural encoding, or task interference during training. ProteinAligner was developed to bridge these gaps by aligning sequence, structure, and text through a unified contrastive framework that preserves continuous geometric details.

## Graphical abstract

**Figure:**
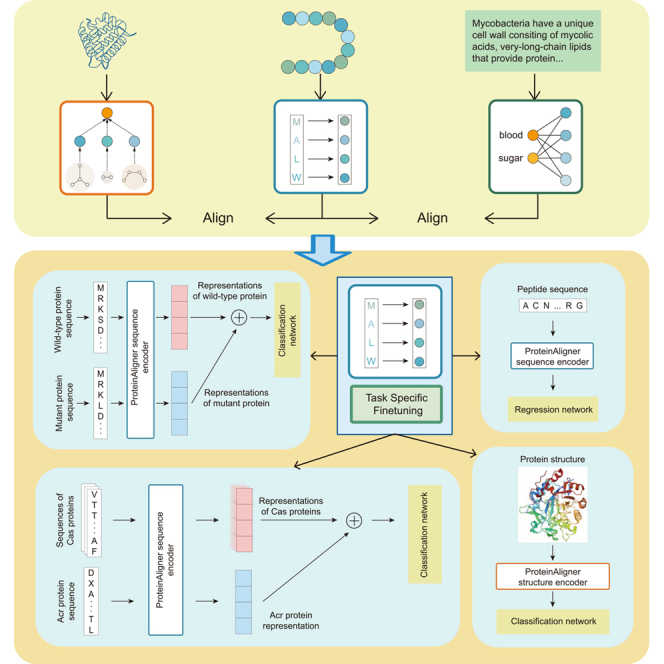

## Teaser

Zhang et al. develop ProteinAligner, a foundation model that teaches AI to understand proteins by simultaneously looking at their genetic sequences, 3D shapes, and scientific descriptions. By merging these diverse perspectives, the model accurately predicts biological functions and properties, offering a powerful tool for drug discovery and biotechnology.

## Introduction

Proteins play a fundamental role in virtually all biological processes. Understanding their functions and properties is central to advancing fields such as drug discovery,^1^ diagnostics,^2^ and biotechnology.^3^ Recent advances in artificial intelligence, particularly in transformer-based models,^4^ have led to the development of protein foundation models capable of learning rich representations from large-scale protein datasets.^5^^,^^6^^,^^6^^,^^6^^,^^7^^,^^8^^,^^9^^,^^10^^,^^11^^,^^12^^,^^13^^,^^14^^,^^15^ These models, particularly protein language models (PLMs),^5^^,^^6^^,^^7^^,^^8^^,^^10^^,^^13^ have shown remarkable success in performing various downstream tasks such as protein function prediction,^16^^,^^17^ property prediction,^18^^,^^19^ structure prediction,^20^^,^^21^ and protein design.^22^^,^^23^ Despite these successes, current PLMs predominantly focus on amino acid sequences while overlooking the wealth of complementary information available in other modalities. Protein structures, for example, provide critical three-dimensional information that is essential for understanding how proteins fold and interact with other molecules, directly influencing their biological functions.^24^ The spatial arrangement of amino acids, which governs interactions such as binding affinities and functional sites, cannot be readily inferred from sequence data alone,^25^^,^^26^ making the integration of structural data crucial for a more comprehensive understanding of protein behavior. Similarly, the vast amount of biological literature contains experimentally validated insights into protein mechanisms, behavior, and interactions that are often context-specific and difficult to infer from sequences or structures alone.^12^^,^^27^ Literature captures critical information about post-translational modifications, protein dynamics in various environments, and interaction networks—details accumulated from years of experimental studies. By incorporating these additional modalities—protein structures and related literature—protein foundation models can move beyond sequence prediction to a more robust, context-aware understanding of protein biology. This multimodal integration has the potential to greatly enhance the representational power of these models, enabling more accurate predictions of protein functions and behaviors in diverse biological scenarios.

Several prior studies^12^^,^^15^^,^^28^^,^^29^^,^^30^ have explored pretraining protein foundation models using pairs of modalities—for example, combining protein sequences with literature texts,^12^ or protein sequences with structural information.^28^ ProteinCLIP^15^ adopts a dual-encoder architecture, aligning protein sequences with functional annotations via contrastive learning in a shared latent space, using a pretrained sequence encoder and a transformer-based text encoder. ProtCLIP^31^ builds upon this by introducing three types of contrastive objectives: (1) sequence-text alignment, (2) intra-sequence consistency across overlapping fragments, and (3) prototype-based alignment for known functional regions. ProtST^12^ also uses contrastive learning between sequences and texts, incorporating curriculum-based negative sampling and a margin-based loss. ESM-S^28^ extends ESM-2^20^ by injecting structural information through fine-tuning on a remote homology detection task. It predicts fold classes directly from sequence embeddings, thereby enriching the model with implicit structural knowledge. However, pretraining on all three modalities remains largely underexplored.

Recently, two works—ESM-3^29^ and ProTrek^30^—conducted independently and concurrently with ours, have leveraged all three modalities for pretraining. ESM-3^29^ integrates amino acid sequences, 3D structural coordinates, and textual annotations by converting each modality into a unified token track processed jointly by a transformer backbone. During pretraining, it employs a masked language modeling (MLM) objective in which tokens across all modalities are randomly masked, and the model is trained to predict the missing elements, thereby learning cross-modal representations. ProTrek^30^ encodes sequences, structures, and functional text using three separate encoders whose outputs are projected into a shared latent space. Its training strategy combines bidirectional InfoNCE^32^ losses with MLM objectives applied separately to the sequence and structure modalities.

ESM-3 exhibits two major limitations. First, its pretraining relies exclusively on masked token prediction and does not incorporate contrastive learning,^32^^,^^33^^,^^34^ which limits its ability to align biologically equivalent inputs such as protein sequences, structures, and functional annotations. This absence of contrastive supervision can lead to fragmented and less transferable representations that perform poorly on tasks requiring integrated biological reasoning. Second, ESM-3 compresses structural information into discrete tokens using a VQ-VAE^35^ encoder, introducing quantization noise that discards fine-grained biophysical details such as side-chain orientations, solvent accessibility, and subtle backbone perturbations. Similarly, ProTrek also exhibits key limitations. Although it combines masked token prediction and contrastive learning during pretraining, this multitask setup can create optimization conflicts due to competing training signals,^36^^,^^37^^,^^38^^,^^39^ leading to unstable convergence and suboptimal alignment across modalities. In addition, ProTrek discretizes protein structures into tokens using Foldseek,^40^ resulting in the loss of detailed geometric information critical for capturing functionally relevant structural features (Figure S5).

To address these limitations, we introduce ProteinAligner, a multimodal pretraining framework that combines protein sequences, structures, and literature text. Our framework aligns these modalities with the protein sequence as the anchor, enabling the model to learn richer and more comprehensive representations of proteins. By integrating diverse protein-related data, ProteinAligner improves the model’s ability to capture intricate biological phenomena, paving the way for more accurate predictions of protein functions and properties. ProteinAligner utilizes three specialized encoders—a sequence encoder, a structure encoder, and a text encoder—to learn representations for each modality. These distinct representations are projected into a shared latent space, enabling direct comparison across modalities. By employing a contrastive alignment strategy,^32^ ProteinAligner uses protein sequences as the anchor to align corresponding structures and textual descriptions, encouraging similar representations for the same protein and dissimilar representations for different proteins. This approach not only maximizes data utilization by allowing pretraining on incomplete modality data but also captures the comprehensive biological insights provided by each modality.

ProteinAligner offers several advantages over existing multimodal protein foundation models. While models such as ProtST, ESM-IF1, ESM-S, ProteinCLIP, and ProtCLIP are limited to two modalities, ProteinAligner simultaneously integrates three: sequence, structure, and functional text. In contrast to ESM-3, which relies on masked token prediction that emphasizes local reconstruction without explicit cross-modal alignment, and ProTrek, which employs a multi-task learning framework prone to task interference, ProteinAligner uses a unified contrastive learning objective that promotes global semantic alignment across modalities and avoids task conflict. Additionally, unlike ESM-3 and ProTrek, which discretize protein structures into tokens and incur quantization errors, ProteinAligner retains continuous structural representations, enabling more precise encoding of geometric features.

ProteinAligner demonstrated superior performance compared to state-of-the-art baselines across various downstream prediction tasks, including predicting pathogenic missense variants, predicting protein thermostability, detecting type I anti-CRISPR activities, identifying potent bioactive peptides, estimating the minimum inhibitory concentration (MIC) of antimicrobial peptides (AMPs), and protein fitness prediction.

## Results

### ProteinAligner overview

**Figure 1:**
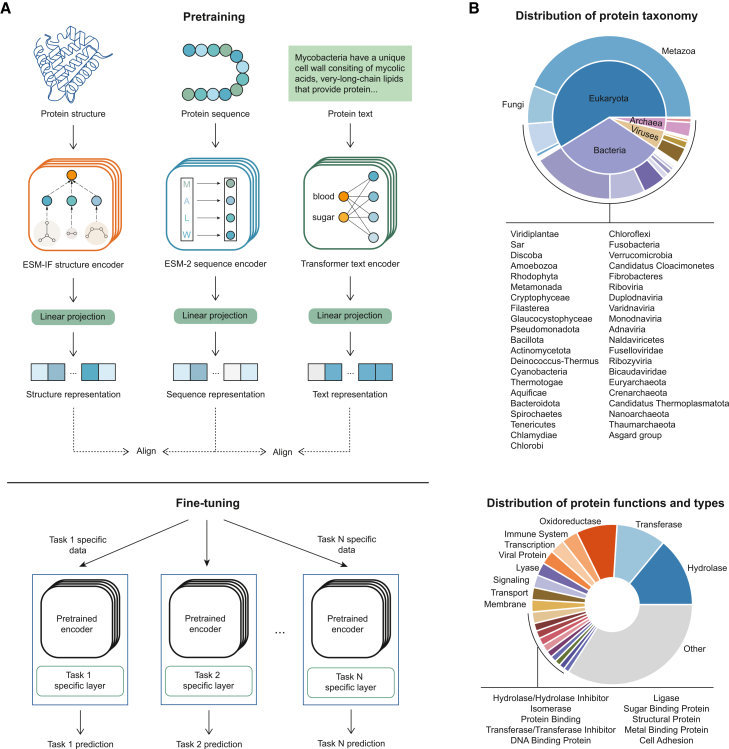
ProteinAligner facilitates multimodal pretraining of protein foundation models by integrating diverse modalities including amino acid sequences, 3D structures, and textual data (A) ProteinAligner consists of three encoders: a protein sequence encoder based on ESM-2 (650M), a protein structure encoder based on ESM-IF1, and a transformer-based protein text encoder. These encoders learn representations for protein sequences, structures, and text, respectively. Modality-specific projection modules then transform these representations into a shared latent space, enabling direct comparison across modalities. Using protein sequences as the anchor, ProteinAligner aligns the other two modalities by minimizing contrastive losses. After pretraining, the encoders can be fine-tuned with task-specific data for various downstream applications. (B) Our curated pretraining data for ProteinAligner spans a diverse range of proteins from various taxonomic groups, functions, and types. The upper chart displays the distribution of protein taxonomy, with the inner ring representing superkingdoms and the outer ring representing kingdoms. The lower chart illustrates the distribution of protein functions and types.

ProteinAligner is a multimodal foundation model for protein representation learning, integrating three distinct modalities: amino acid (AA) sequences, 3D structures, and textual descriptions of proteins. ProteinAligner contains three encoders—a protein sequence encoder, a protein structure encoder, and a text encoder—each dedicated to learning representations for its corresponding modality (Figure 1A). The protein sequence encoder is a PLM that uses the transformer architecture^4^ to extract a representation for the input AA sequence. It represents each AA as a token and employs self-attention^4^ to capture long-range dependencies among AAs. The protein structure encoder utilizes geometric vector perceptron graph neural network (GVP-GNN)^41^ layers for geometric representation learning of the input protein structure, followed by transformer layers that capture long-range interactions between atomic coordinates. The text encoder employs transformer architecture, utilizing self-attention to capture long-range dependencies between language tokens. Specifically, we employed ESM-2 (650M),^20^ a leading PLM, as the protein sequence encoder, and ESM-IF1^9^ as the protein structure encoder. ESM-2 (650M) consists of 33 transformer layers and 650 million parameters, pretrained on 65 million protein sequences. ESM-IF1 features 20 layers and 124 million parameters, pretrained on 12 million computed protein structures and 16,000 experimentally verified structures. The text encoder includes eight transformer layers with a total of 78 million parameters. ProteinAligner uses modality-specific linear projection modules to map the representations extracted by different encoders into a shared latent space with matching dimensions, ensuring that representations from different modalities are directly comparable. ProteinAligner consists of 867 million model parameters in total.

ProteinAligner performs joint pretraining of the three encoders by leveraging a modality alignment strategy, using protein sequences as the anchor modality to align the other two modalities (Figure 1A). Specifically, given a protein sequence and a protein structure, if they correspond to the same protein, ProteinAligner encourages their representations to be similar, and dissimilar otherwise. The same principle applies for protein text and protein sequences, with representations aligned if they refer to the same protein and separated if not. This alignment is accomplished by minimizing contrastive losses^32^^,^^33^ defined on the representations of sequence-structure pairs and sequence-text pairs. ProteinAligner does not require all three modalities to be present simultaneously for each protein in the pretraining data. The alignment can be performed as long as the protein sequence and at least one additional modality—either structure or text—are available. We chose protein sequences as the anchor for alignment because they are the most prevalent data modality in protein databases; nearly every protein has an associated amino acid sequence, whereas information on structures or textual descriptions is often incomplete. By using sequences as the anchor, we can maximize data utilization, ensuring the inclusion of as many proteins as possible in the alignment process. The advantage of tri-modal pretraining in ProteinAligner is demonstrated by a comprehensive ablation study to evaluate the incremental contribution of each modality (Figure S2). With pretrained encoders in place, they can be fine-tuned on task-specific data to handle a variety of downstream tasks. During this process, the encoders are integrated with task-specific modules, creating models that are customized for specific prediction tasks. For all downstream tasks, we kept the pre-trained encoders completely frozen. Only the parameters of the newly initialized, task-specific prediction heads were updated during training without any parameter-efficient fine-tuning techniques. Crucially, such a fine-tuning process was applied to all baseline models for fair comparisons.

We curated a large-scale pretraining dataset for ProteinAligner by integrating data from the UniProtKB/Swiss-Prot^42^ and RCSB PDB^43^ databases. The dataset consists of 290,480 proteins, each with an amino acid sequence and a corresponding textual description. 133,726 of them are also associated with protein structures. In total, the dataset contains 133,726 sequence-structure pairs and 290,480 sequence-text pairs. The textual descriptions provide information about the proteins’ functions. Both the structures and the functional descriptions were experimentally validated and reviewed by domain experts. Figure 1B shows the distribution of protein taxonomy, functions, and types in the dataset. We conducted a comprehensive overlap analysis between the downstream task test sets and the pretraining corpus using BLASTp.^44^ No sequence in any downstream test set showed detectable similarity to sequences in the pretraining data, confirming the absence of data overlap.

### ProteinAligner predicts pathogenic missense variants

Pathogenic missense variants refer to specific types of genetic mutations where a single nucleotide change in a DNA sequence results in the substitution of one amino acid for another in the corresponding protein.^45^ This change can disrupt the protein’s normal function, potentially leading to diseases or disorders. In the context of pathogenicity, these variants are considered harmful because they alter the protein’s structure or function in a way that impairs biological processes. Depending on the protein’s role, this can lead to a variety of outcomes, from minor effects to severe genetic disorders, such as cystic fibrosis, sickle cell disease, or certain forms of cancer. Identifying and characterizing pathogenic missense variants is crucial in genetic research and clinical diagnostics for understanding inherited diseases and developing targeted treatments.

**Figure 2:**
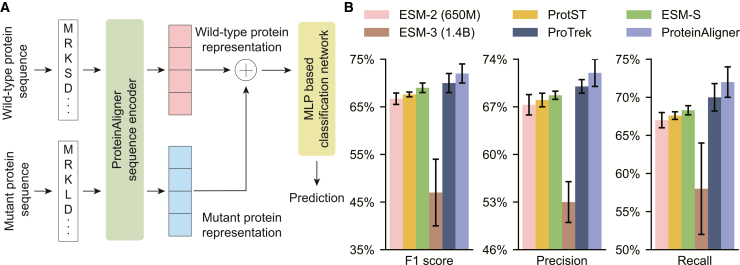
ProteinAligner excels in predicting pathogenic missense variants compared to existing protein foundation models (A) Model architecture used to fine-tune the pretrained ProteinAligner sequence encoder for this task. (B) ProteinAligner achieves higher performance than ESM-2 (650M), ProtST, ESM-S, ESM-3 (1.4B), and ProTrek, as measured by F1 score, precision, and recall. Data are represented as “mean ± standard deviation” over three independent runs. The error bars indicate the standard deviation across three independent runs with different random seeds.

The inputs for this task are two protein sequences: the wild-type sequence (before mutation) and the mutant sequence (after mutation). We employed the sequence encoder in ProteinAligner to extract representation vectors for both proteins. These vectors were then concatenated and passed through a multi-layer perceptron to predict whether the mutant protein is pathogenic (Figure 2A). We used 200 labeled examples from the VariPred^46^ dataset, with 100 examples allocated for training and the remaining 100 for testing.

We benchmarked ProteinAligner against five state-of-the-art protein foundation models: (1) ESM-2 (650M),^20^ a PLM pretrained solely on amino acid sequences; (2) ProtST,^12^ which uses contrastive learning to align protein sequences with functional texts; (3) ESM-3 (1.4B),^29^ pretrained jointly on sequences, structures, and functional annotations using a MLM objective across all three modalities; (4) ProTrek,^30^ trained on sequences, structures, and texts via a multi-task framework combining masked modeling and contrastive learning; and (5) ESM-S,^28^ which incorporates 3D structural priors into the ESM-2 model via remote homology supervision. We did not include direct comparisons with ProteinCLIP^15^ and ProtCLIP,^31^ as both follow a similar two-modality contrastive framework as ProtST. Model performance was assessed using F1-score, precision, and recall. For this and all other downstream tasks, we retrained every model five times using different random initializations of the task-specific prediction head. We then reported the mean and standard deviation of the evaluation metrics across the five runs. We also compared ProteinAligner with the retrieved sequence augmentation (RSA) approach (Figure S3) to evaluate whether retrieval-based sequence augmentation provides comparable benefits.

ProteinAligner outperformed all baseline methods across F1 score, precision, and recall (Figure 2B). The corresponding *p* values from two-sided *t* tests—computed based on five repeated runs under identical settings with variation only in random seed initialization—comparing ProteinAligner to ESM-S, ESM-3, and ProTrek on F1 score are 0.045, <0.01, and 0.037, respectively. All *p* values fall below the 0.05 threshold, indicating that the F1 score improvements achieved by ProteinAligner are statistically significant. Despite ESM-3’s substantially larger scale—with 1.4 billion parameters and a pretraining corpus comprising 2.78 billion natural protein sequences, which are augmented to over 771 billion sequence tokens, in addition to 236 million structure tokens and 539 million function annotation tokens—its performance remains markedly below that of ProteinAligner. ESM-3 achieves only 0.53 precision, 0.58 recall, and 0.47 F1 score, compared to ProteinAligner’s scores of 0.72, 0.72, and 0.72, respectively, despite ProteinAligner’s considerably smaller model size and pretraining dataset.

### ProteinAligner predicts protein thermostability

Protein thermostability refers to a protein’s ability to maintain its structure and function when exposed to elevated temperatures.^47^ This characteristic is critical because proteins typically lose their functional shape, or denature, at high temperatures, rendering them ineffective. Thermostability is an important factor in various biological processes and industrial applications. For instance, enzymes with high thermostability are essential in industries such as biotechnology and pharmaceuticals, where reactions often require high temperatures for optimal efficiency. Predicting protein thermostability allows researchers to design or engineer proteins that can withstand challenging conditions, improving their functionality and longevity. Additionally, thermostable proteins are valuable in drug design, as they tend to have better shelf lives and performance under physiological conditions. Accurate predictions of thermostability are crucial for advancing protein engineering and enhancing the reliability of proteins in various applications.

**Figure 3:**
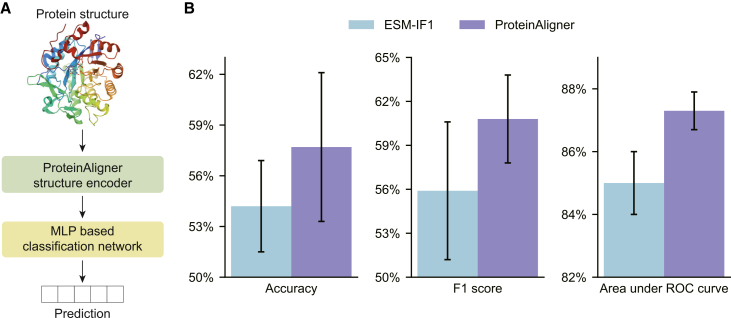
ProteinAligner demonstrates superior performance in predicting protein thermostability compared to existing protein foundation models (A) Model architecture used for fine-tuning the pretrained ProteinAligner structure encoder for this task. (B) ProteinAligner outperforms ESM-IF1, achieving higher accuracy, F1 score, and area under ROC curve. Data are represented as “mean ± standard deviation” over three independent runs. The error bars indicate the standard deviation across three independent runs with different random seeds.

Unlike the previous task, this task takes the 3D structures of proteins, specifically their atomic coordinates, as input. The 3D structure of each protein was processed through ProteinAligner’s structure encoder, generating a representation vector. This vector was then passed through a multi-layer perceptron to predict the protein’s thermostability class (Figure 3A). We employed the HP-S^2^C5 dataset,^48^ which comprises 1,040 proteins spanning five thermostability classes: hyperthermophilic (above 75°C), thermophilic (45°C–75°C), mesophilic (25°C–45°C), psychrophilic (5°C–25°C), and cryophilic (−20°C to 5°C). Nine hundred thirty-six proteins were used for training and 104 for testing. We compared ProteinAligner with ESM-IF1,^9^ a protein structure encoder pretrained on both protein structures and sequences. We used accuracy, F1 score, and area under ROC curve as evaluation metrics. ProteinAligner outperformed ESM-IF1 (Figure 3B), achieving an F1 score of 0.608 compared to 0.559, and an accuracy of 0.577 compared to 0.542.

### ProteinAligner detects type I anti-CRISPR activities

We evaluated the effectiveness of ProteinAligner in detecting type I anti-CRISPR (Acr) activities. Acr proteins are produced by certain viruses, such as bacteriophages, or mobile genetic elements to inhibit the type I CRISPR-Cas immune system in bacteria and archaea.^49^ The CRISPR-Cas system functions as an adaptive immune mechanism in these microorganisms, recognizing and cleaving foreign DNA from viral invaders. In type I systems, which involve multi-subunit Cas proteins, Acr proteins disrupt this defense by preventing Cas proteins from binding to target DNA or carrying out their cleavage functions. Understanding and detecting these Acr activities is crucial for controlling CRISPR-Cas systems in genetic engineering and leveraging bacteriophages to combat antimicrobial resistance.

**Figure 4:**
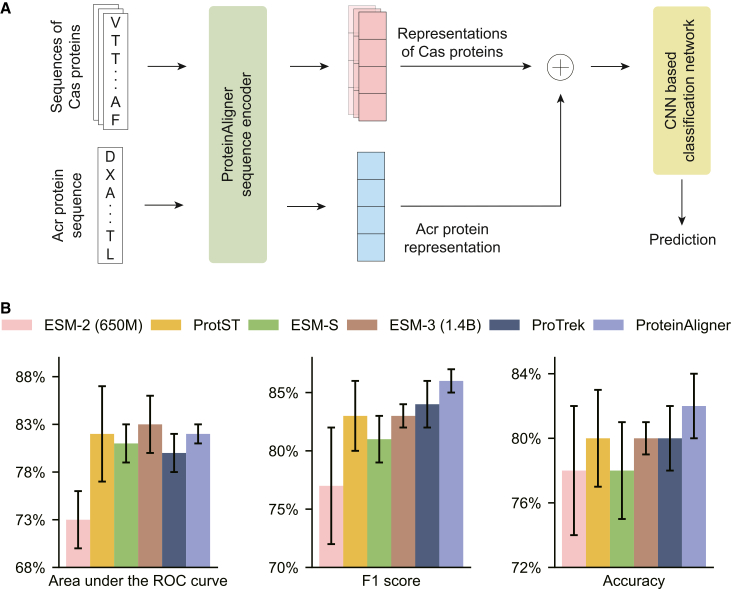
ProteinAligner demonstrates strong performance in detecting type I anti-CRISPR activities (A) Model architecture for fine-tuning the pretrained ProteinAligner sequence encoder for predicting type I anti-CRISPR activities. (B) ProteinAligner outperformed all baselines in terms of F1 score and accuracy. For area under the ROC curve (AUC), it achieved the second-highest performance, slightly trailing ESM-3 (1.4B). Data are represented as “mean ± standard deviation” over three independent runs. The error bars indicate the standard deviation across three independent runs with different random seeds.

Given the amino acid sequences of an Acr protein and a set of Cas proteins from a CRISPR-Cas system, we employed ProteinAligner’s pretrained sequence encoder to extract representation vectors for each protein. These vectors were then input into a convolutional neural network (CNN)-based classification module to predict whether the Arc protein could inhibit the CRISPR-Cas system (Figure 4A). We utilized the Acr-CRISPR-Cas inhibition dataset^49^ for experiments, which comprises 227 pairs of Acr proteins and CRISPR-Cas systems, including 132 experimentally verified positive pairs (Acr inhibits CRISPR-Cas) and 95 negative pairs (Acr does not inhibit CRISPR-Cas). The dataset was randomly split into training and test sets in an 8:2 ratio.

ProteinAligner outperformed all baselines in terms of accuracy. For area under the ROC curve (AUC), it achieved the second-highest performance, slightly trailing ESM-3 (1.4B). In terms of F1 score, the *p* values for comparisons between ProteinAligner and ESM-S, ESM-3, and ProTrek are 0.018, 0.011, and 0.033, respectively. All values fall below the 0.05 threshold, indicating that the improvements achieved by ProteinAligner over these baselines are statistically significant. Additionally, we constructed a strictly non-overlapping test set with remote-homology filtering to assess the generalization ability of ProteinAligner (Figure S2).

### ProteinAligner identifies potent bioactive peptides

Bioactive peptides are short chains of amino acids with specific biological activities.^50^ They play critical roles in regulating physiological processes, including immune function, metabolism, and cardiovascular health. Identifying bioactive peptides is important because they offer significant potential for developing new therapeutic agents and functional foods. These peptides can serve as natural, targeted treatments with fewer side effects compared to traditional drugs, and their discovery can lead to advancements in both medical applications and nutrition, benefiting public health and disease prevention efforts.

Given the amino acid sequence of a peptide, we employed ProteinAligner’s protein sequence encoder to extract a representation vector, which was subsequently input into a CNN-based classification head to predict whether the peptide has a specific bioactivity. We examined seven distinct bioactivities, including inhibition of dipeptidylpeptidase IV (DPP-IV),^51^ modulation of brain activity,^52^ antiviral properties,^53^ antioxidant activity,^54^ umami taste induction,^55^ blood-brain barrier penetration,^56^ and T cell immune response induction.^57^ Given that a peptide can exhibit multiple bioactivities concurrently, we approached each bioactivity prediction as a binary classification task, avoiding the use of a multi-class model that would assign the peptide to a single category. Separate datasets were used for each bioactivity (“methods”). The evaluation metrics for this task included accuracy (ACC), balanced accuracy (BACC),^58^ sensitivity (SN), specificity (SP), Matthews correlation coefficient (MCC),^59^ and the area under the ROC curve (AUC).

**Figure 5:**
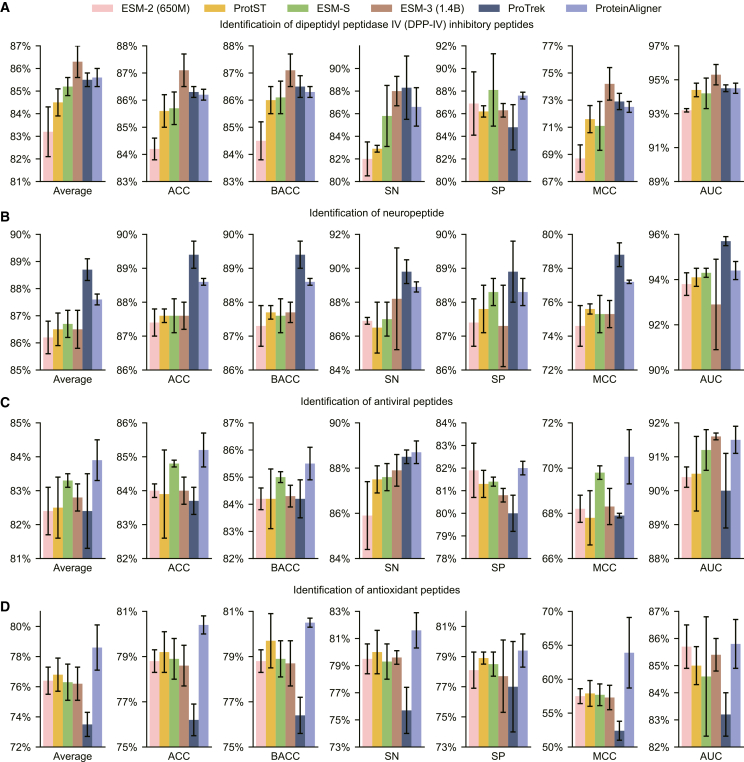
ProteinAligner demonstrates superior performance in identifying potent bioactive peptides Across four tasks—predicting inhibition of dipeptidylpeptidase IV (DPP-IV) (A), modulation of brain activity (B), antiviral properties (C), and antioxidant activity (D)—ProteinAligner outperformed ESM-2 (650M), ProtST, and ESM-S in all tasks, and surpassed ESM-3 (1.4B) and ProTrek in three out of four tasks. Model performance was evaluated using accuracy (ACC), balanced accuracy (BACC), sensitivity (SN), specificity (SP), Matthews correlation coefficient (MCC), and the area under the ROC curve (AUC). “Average” refers to the mean value across these six metrics. Data are represented as “mean ± standard deviation” over three independent runs. The error bars indicate the standard deviation across three independent runs with different random seeds.

**Figure 6:**
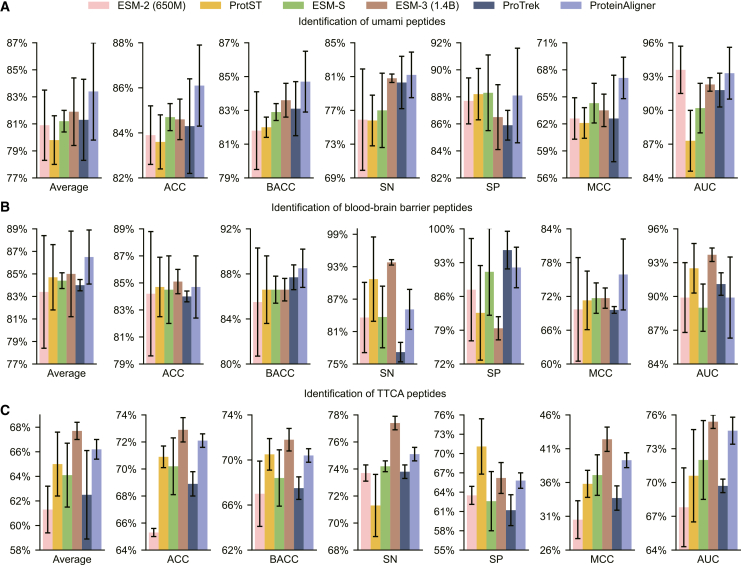
ProteinAligner also demonstrates superior performance in three additional tasks related to bioactive peptide identification Specifically, in predicting umami taste induction (A), blood-brain barrier penetration (B), and T cell immune response induction (C), ProteinAligner outperformed ESM-2 (650M), ProtST, ESM-S, and ProTrek across all tasks, and exceeded ESM-3 (1.4B) in two out of the three tasks. Model performance was assessed using accuracy (ACC), balanced accuracy (BACC), sensitivity (SN), specificity (SP), Matthews correlation coefficient (MCC), and the area under the ROC curve (AUC). “Average” refers to the mean value across these six metrics. Data are represented as “mean ± standard deviation” over three independent runs. The error bars indicate the standard deviation across three independent runs with different random seeds.

ProteinAligner outperformed the baselines in most cases (Figures 5 and 6). For example, in terms of average performance, ProteinAligner outperformed ESM-2, ProtST, and ESM-S in all seven tasks, outperformed ProTrek in six of the seven tasks, and outperformed ESM-3 in five out of the seven tasks.

### ProteinAligner predicts the MIC of AMPs

AMPs are short chains of amino acids that serve as a crucial part of the innate immune response in many organisms, exhibiting broad-spectrum activity against bacteria, viruses, fungi, and even cancer cells.^60^ They function by disrupting microbial membranes, leading to cell death, and are considered potential alternatives to conventional antibiotics, especially in the face of rising antibiotic resistance. The MIC is the lowest concentration of an antimicrobial agent, such as an AMP, that prevents visible microbial growth. Accurately predicting the MIC values of AMPs is essential as it allows for the optimization of peptide design for therapeutic use, minimizes potential toxicity, and helps in the early-stage screening of effective peptides before *in vitro* or *in vivo* testing. This predictive capability is vital for accelerating the development of AMPs as a novel class of antimicrobial agents in clinical applications.

**Figure 7:**
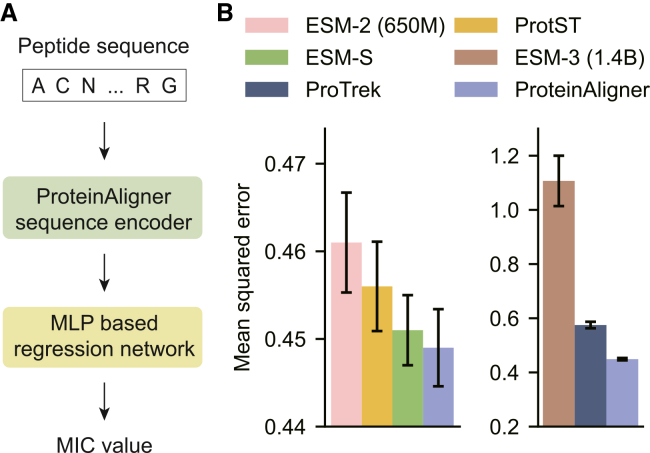
ProteinAligner outperforms existing protein foundation models in predicting the MIC of antimicrobial peptides (A) Model architecture designed for this prediction task. (B) ProteinAligner achieved a lower mean squared error than ESM-2 (650M), ProtST, ESM-S, ESM-3 (1.4B), and ProTrek. Data are represented as “mean ± standard deviation” over three independent runs. The error bars indicate the standard deviation across three independent runs with different random seeds.

Given the amino acid sequence of a peptide, we applied ProteinAligner’s protein sequence encoder to extract a representation vector, which was then fed into a multi-layer perceptron-based regression module to predict the MIC of the peptide against a specific pathogen (Figure 7A). We focused on *Escherichia coli* (*E. coli*), a gram-negative bacterium. We utilized the dataset from,^61^ comprising 3,695 training and 924 testing examples. Mean squared error was used as the evaluation metric. ProteinAligner achieved lower prediction error compared to ESM-2, ProtST, ESM-S, ESM-3, and ProTrek (Figure 7B). ESM-3 had the highest error among all methods, with a value of 1.1—substantially higher than ProteinAligner’s error of 0.449. This observation aligns with the findings reported in Zhao et al.^62^ And, we conducted zero-shot evaluation to assess the generalization capability of ProteinAligner without task-specific fine-tuning (Figure S1).

## Discussion

ProteinAligner introduces a comprehensive approach to protein representation learning by integrating amino acid sequences, continuous 3D structures, and literature texts into a unified framework. This multimodal design allows the model to capture complementary information from each modality, providing a richer and more holistic understanding of proteins. By employing contrastive alignment, ProteinAligner learns representations that incorporate structural and functional attributes alongside contextual knowledge from experimental literature, enabling superior performance across a range of challenging protein-related tasks (Figures 2, 3, 4, 5, 6, and 7). Its ability to bridge critical gaps in existing models demonstrates its potential for advancing research in protein biology, drug development, and biotechnology, highlighting the importance of multimodal frameworks in addressing complex biological challenges.

The superior performance of ProteinAligner over ESM-2 (Figures 2, 4, 5, 6, and 7) can be primarily attributed to the differences in their pretraining strategies. ESM-2 is pretrained exclusively on large-scale protein sequences using a MLM objective, which allows it to capture local and global sequence patterns but lacks exposure to structural or functional context. In contrast, ProteinAligner adopts a multimodal pretraining framework that jointly leverages protein sequences, protein 3D structures, and textual descriptions of protein function. This integration of multiple biologically relevant modalities enables ProteinAligner to learn richer and more biologically grounded representations. Structural data encode critical spatial and physicochemical properties, such as residue-residue proximity, binding pocket geometry, and overall protein folding, which are not directly inferable from sequence alone. Additionally, functional textprovides high-level semantic information about biological roles, molecular mechanisms, and cellular processes, complementing the syntactic patterns learned from sequence and the geometric insights from structure. By aligning representations across sequence, structure, and function during pretraining, ProteinAligner is able to internalize correspondences between syntax, shape, and semantics in protein biology. This cross-modal alignment enhances its ability to generalize across a broad range of downstream prediction tasks, including those requiring inference of higher-order biological properties. As a result, ProteinAligner consistently outperforms ESM-2, especially in tasks where structural context or functional semantics are essential for accurate prediction. This highlights the advantage of incorporating multimodal biological knowledge during pretraining and underscores the importance of moving beyond sequence-only approaches when modeling protein function and behavior.

ProteinAligner outperforms ProtST (Figures 2, 4, 5, 6, and 7) largely due to the incorporation of structural information during pretraining. While both models utilize protein sequences and functional text, ProtST does not leverage 3D structural data, limiting its ability to capture the spatial and physicochemical context critical to understanding protein function. ProteinAligner’s multimodal framework integrates sequence, structure, and function, enabling it to align these three complementary modalities and learn more holistic protein representations. Structural information provides key insights into residue interactions, conformational flexibility, and binding site geometry—factors that often underpin functional behavior but are not readily apparent from sequence or text alone. By incorporating this additional modality, ProteinAligner can better generalize to tasks that require nuanced understanding of protein conformation or interactions. Moreover, the joint modeling of structure with sequence and text enables the model to associate semantic functional descriptors with both linear and spatial features of proteins, which enhances interpretability and predictive power. This comprehensive representation explains ProteinAligner’s consistent advantage over ProtST across a range of downstream tasks.

The performance advantage of ProteinAligner over ESM-IF1 and ESM-S (Figures 2, 4, 5, 6, and 7) can be attributed to its incorporation of functional text during pretraining, in addition to protein sequences and structures. While ESM-IF1 and ESM-S capture rich structural and sequential patterns, they are not exposed to explicit functional semantics, which limits their ability to align molecular features with biological meaning. In contrast, ProteinAligner leverages textual descriptions of protein function as an additional modality, enabling it to associate structural and sequence features with high-level functional attributes described in natural language. This grounding in functional text enhances the model’s ability to recognize biologically relevant patterns that may not be evident from sequence or structure alone. For instance, two proteins with divergent sequences or conformations may share a similar function—a relationship that functional text helps to bridge. By jointly pretraining on sequence, structure, and function, ProteinAligner develops semantically informed representations that improve generalization across diverse downstream tasks, particularly those involving function prediction or annotation transfer. This integrated approach gives ProteinAligner an edge over structure-sequence-only models like ESM-IF1 and ESM-S, which lack direct exposure to the linguistic and conceptual framing of protein function.

ProteinAligner outperforms ESM-3 in most downstream tasks and across most evaluation metrics (Figures 2, 5, 6, and 7), which can be primarily attributed to two key factors. First, ESM-3’s pretraining strategy relies exclusively on masked token prediction and does not incorporate a contrastive learning objective. Without contrastive supervision, the model is not explicitly encouraged to bring representations of biologically equivalent inputs—such as a protein’s sequence and its corresponding structure or functional annotation—closer together in the embedding space. As a result, ESM-3 may fail to establish coherent cross-modal alignments, leading to fragmented and less transferable representations that underperform on tasks requiring integrated biological reasoning, such as pathogenic variant classification (Figure 2) and MIC prediction (Figure 7). In contrast, our method employs contrastive learning to align modalities explicitly, encouraging the model to capture shared semantics and fine-grained relations between different modalities, which significantly enhances generalization to downstream tasks. Second, the representational fidelity of ESM-3 is further limited by quantization noise introduced by its compression of each residue’s continuous 3D neighborhood into a single discrete “structure token” using a VQ-VAE^35^ encoder. This discretization process discards critical biophysical information—such as side-chain orientations,^63^ solvent accessibility,^64^ and sub-Ångström backbone perturbations^65^^,^^66^—that are essential for capturing fine structural determinants of protein behavior. In contrast, our method’s structure encoder, based on the inverse-folding model ESM-IF1,^9^ operates directly on full backbone atomic coordinates without quantization. By leveraging rotation- and translation-equivariant geometric vector perceptron (GVP) graph layers,^41^ ProteinAligner inherits a strong geometric inductive bias that faithfully captures the spatial symmetries of protein folds.^41^ Aligning these geometry-aware structural embeddings with protein sequence embeddings propagates physically meaningful constraints that ESM-3 is unable to exploit, as its quantized structure consists of discretized tokens lacking built-in equivariance.

ProteinAligner also outperforms ProTrek in most tasks (Figures 2, 4, 5, 6, and 7), likely due to two main factors. First, while ProTrek employs a combination of masked token prediction and contrastive learning during pretraining, this multitask setup introduces inherent optimization conflicts. MLM encourages the model to focus on local contextual reconstruction, optimizing for token-level accuracy. In contrast, contrastive learning promotes global alignment between modalities by pulling together semantically related representations and pushing apart unrelated ones. These two objectives often operate at different granularities and may impose competing gradient signals during training, which can lead to unstable convergence, diminished alignment quality, and suboptimal representation learning. Empirical studies have shown that when multitask objectives are not carefully balanced, they can interfere with each other, reducing the effectiveness of both.^36^^,^^37^^,^^38^ ProteinAligner avoids this issue by adopting a streamlined pretraining objective based solely on contrastive learning. This choice allows the model to concentrate on learning globally consistent, modality-aligned representations without the interference of reconstruction-based losses, resulting in more coherent and transferable embeddings for downstream tasks. Second, ProteinAligner’s structural encoder preserves continuous 3D geometry using a GVP^41^-based architecture, whereas ProTrek represents protein structures by discretizing them into tokens using Foldseek.^40^ This discretization introduces a loss of fine-grained spatial information—such as torsion angles, side-chain orientation, and atomic-level packing—that are crucial for modeling functionally relevant features.

ProteinAligner’s ability to perform a wide range of prediction tasks presents promising applications across biology, drug discovery, and medicine. In drug discovery, ProteinAligner’s accurate identification of bioactive peptides, such as DPP-IV inhibitors (Figure 5A), is particularly relevant for developing treatments for metabolic disorders like diabetes. Its capability to predict AMP properties, such as MIC (Figure 7B), is critical for advancing new antimicrobial therapies, especially in addressing the challenge of antibiotic-resistant pathogens. This has important implications for the global fight against antimicrobial resistance. The model’s ability to detect type I anti-CRISPR activities supports the design of more efficient and precise CRISPR-based tools for both research and therapeutic applications (Figure 4B). Anti-CRISPR systems could be used to enhance the safety of gene editing by mitigating off-target effects or enabling reversible gene modifications. In precision medicine, the prediction of pathogenic missense variants aids in the early detection and diagnosis of genetic disorders. By identifying harmful mutations that may lead to diseases, ProteinAligner can contribute to personalized treatment strategies, improving patient outcomes (Figure 2B). Protein fitness prediction accelerates enzyme engineering and therapeutic protein design by pinpointing mutations that enhance catalytic efficiency, stability, or specificity (Figure S1). Additionally, ProteinAligner’s accurate prediction of protein thermostability is vital for protein engineering, biopharmaceutical development, and industrial biotechnology, where stable proteins are necessary for drug formulations and biocatalysts (Figure 3B). Overall, ProteinAligner’s diverse prediction capabilities position it as a valuable tool that can accelerate innovation in multiple fields, enabling faster therapeutic discoveries, more precise gene-editing tools, and advancements in personalized medicine and protein engineering.

The significance of ProteinAligner lies primarily in its architectural and conceptual design, specifically, in how contrastive alignment is applied across three heterogeneous biological modalities: protein sequences, 3D structures, and textual descriptions. Unlike prior models that employ contrastive learning in two-modality settings (e.g., sequence-text or sequence-structure), ProteinAligner introduces a tri-modal alignment framework that uses protein sequences as a unifying anchor to jointly align both structural and textual representations within a shared latent space. This design enables implicit structure-text coupling through the sequence anchor, allowing the model to integrate geometric and semantic information efficiently without requiring all three modalities to be simultaneously available for each protein. In addition, ProteinAligner preserves continuous 3D geometric features through a GVP-GNN-based structure encoder rather than discretizing structural inputs into tokens as done in ESM-3 and ProTrek, thereby maintaining fine-grained spatial information during alignment. The combination of these designs—anchor-based tri-modal alignment and continuous geometric encoding—constitutes the methodological advance of our work beyond existing contrastive frameworks.

Future work on ProteinAligner could focus on several key directions to further enhance its performance and applicability. One promising area is the incorporation of additional modalities, such as protein-protein interaction networks and post-translational modifications. These additional data sources could provide deeper insights into protein behavior and interactions, leading to even more robust and comprehensive protein representations. Another direction for future work is to improve the model’s ability to handle incomplete or noisy data by developing more sophisticated alignment strategies that better tolerate inconsistencies between modalities. Enhancing the interpretability of ProteinAligner’s predictions is also a critical area for future research, which could involve incorporating explainability techniques to make the model’s decision-making process more transparent, particularly in cases where sequence, structure, and text data converge. Lastly, expanding ProteinAligner’s applications beyond protein function and property prediction—such as protein design and structure prediction—could broaden its impact across a wide range of biological and biomedical challenges.

### Limitations of the study

Despite the advantages of ProteinAligner, the model has several limitations. One of the key challenges is the dependency on high-quality structural and textual data, which is not always available for all proteins. While ProteinAligner can perform pretraining even when only sequences and one additional modality (either structure or text) are present, the absence of full multimodal data for many proteins can limit the model’s ability to learn comprehensive representations. Additionally, ProteinAligner’s reliance on contrastive loss for modality alignment may not fully capture subtle biological nuances in cases where sequence, structure, and text data are not perfectly aligned. Another limitation is the computational cost associated with training multimodal models, especially when dealing with large-scale protein datasets that involve high-dimensional structural information and large text corpora. Finally, while ProteinAligner improves upon previous models by integrating structure, sequence, and text, it still does not account for other potentially informative modalities, such as protein-protein interactions or functional annotations from various databases, which could further enhance its predictive capabilities.

## Resource availability

### Lead contact

Requests for further information and resources should be directed to and will be fulfilled by the lead contact, Pengtao Xie (p1xie@ucsd.edu).

### Materials availability

This study did not generate new materials.

### Data and code availability

• The FASTA and PDB datasets are publicly available in UniProtKB Swiss-Prot and RCSB PDB, respectively. The FASTA and PDB entries for protein sequences and structures in the pretraining data, along with their textual descriptions, are available at https://doi.org/10.5281/zenodo.18806818. All the data used in downstream tasks are also publicly available. The data for predicting the pathogenicity of missense variants are available at VariPred. The data used in thermostability prediction are available at HotProtein. The data used in type I anti-CRISPR activity detection are available at AcrTransAct. The data used in peptide bioactivity prediction are available at UniDL4BioPep. The data for predicting the minimum inhibitory concentration (MIC) of antimicrobial peptides are available at DeepAMP.
• The source code for ProteinAligner pretraining is available at https://doi.org/10.5281/zenodo.18806818. Additionally, links to the code for downstream tasks can be found in the README file.
• Any additional information required to reanalyze the data reported in this paper is available from the lead contact upon request.

## Acknowledgments

This work was funded by P.X. via grants NSF IIS2405974, NSF IIS2339216, 10.13039/100000002NIH R35GM157217, and 10.13039/100000002NIH R21GM154171. The authors thank all members of the lab for their support.

## Author contributions

Conceptualization, L.Z., H.G., and P.X.; methodology, L.Z., H.G., and P.X.; investigation, L.Z., H.G., E.X., and P.X.; writing – original draft, L.Z., H.G., and P.X.; writing – review & editing, L.Z., H.G., L.S., Y.S.K., D.S., H.R., D.G., E.V., M.G., W.W., T.I., E.X., and P.X.; funding acquisition, P.X.; resources, E.X. and P.X.; supervision, E.X. and P.X.

## Declaration of interests

T.I. is a cofounder, member of the advisory board, and has an equity interest in Data4Cure and Serinus Biosciences.

## Declaration of generative AI and AI-assisted technologies in the writing process

During the preparation of this work, the authors used ChatGPT in order to polish writing and fix grammar mistakes. After using this tool or service, the authors reviewed and edited the content as needed and take full responsibility for the content of the publication.

## STAR★Methods

### Key resources table

**Table:** 

REAGENT or RESOURCE	SOURCE	IDENTIFIER
**Deposited data**		

Pretraining data	This paper	https://doi.org/10.5281/zenodo.18806818
UniProtKB/Swiss-Prot	UniProt	https://www.uniprot.org/
RCSB Protein DataBank (PDB)	PDB	https://www.rcsb.org/
VariPred dataset	Weining Lin et al.	https://github.com/wells-jude/VariPred
HP-S^2^C5 dataset (HotProtein)	Tianlong Chen et al.	https://github.com/VITA-Group/HotProtein
Acr-CRISPR-Cas inhibition dataset	Moein Hasani et al.	https://github.com/USask-BINFO/AcrTransAct
UniDL4BioPep peptide datasets	Zhenjiao Du et al.	https://github.com/dzjxzyd/UniDL4BioPep
Gram-negative AMP dataset	Amir Pandi et al.	https://github.com/amirpandi/Deep_AMP
ProteinGym benchmark	Pascal Notin et al.	https://proteingym.org/

**Software and algorithms**		

ProteinAligner (Source code)	This paper	https://doi.org/10.5281/zenodo.18806818
ESM-2 (650M) sequence encoder	Zeming Lin et al.	https://github.com/facebookresearch/esm
ESM-IF1 structure encoder	Chloe Hsu et al.	https://github.com/facebookresearch/esm
PyTorch Distributed (DDP)	PyTorch	https://pytorch.org/

### Method details

#### Dataset preprocessing

The pretraining data for ProteinAligner was sourced from the UniProtKB/Swiss-Prot^42^ and RCSB PDB^43^ databases. UniProtKB/Swiss-Prot is a well-curated repository containing high-quality protein sequences across a wide variety of organisms, along with detailed annotations on protein functions and properties. We utilized version UniProt 2023_02, which was released on May 2, 2023. The RCSB PDB database offers a comprehensive collection of experimentally determined 3D protein structures, derived from methods such as X-ray crystallography, nuclear magnetic resonance (NMR) spectroscopy, and cryo-electron microscopy (cryo-EM). It includes protein structures from a wide range of proteins, such as enzymes, receptors, and antibodies, originating from diverse organisms. From these databases, we collected sequence-text pairs and sequence-structure pairs. The sequence-text pairs were sourced from UniProtKB/Swiss-Prot. We first obtained a collection of protein entries from Swiss-Prot that included textual descriptions of their functions, by filtering for entries where the commentType field was set to ‘Function’. We then retrieved the corresponding sequence for each protein in this collection. Specifically, we accessed the UniProt ID from the primaryAccession field and used it to retrieve the corresponding protein FASTA file from the UniProt website, which contains the protein’s sequence. We downloaded all available PDB files from the May 2, 2023 dataset release,^43^ comprising 200,734 experimentally determined protein structures. We then employed the UniProt ID mapping tool (https://www.uniprot.org/id-mapping) to link the structures in the PDB files to their corresponding amino acid sequences in the FASTA files. To address memory constraints during pretraining, we excluded protein sequences longer than 300 residues, yielding 133,726 sequence-structure pairs and 290,480 sequence-text pairs. And, we investigated whether incorporating high-confidence predicted structures improves model performance, we expanded the pretraining corpus by adding 119,485 sequence–structure pairs from the AlphaFold DB and re-pretrained ProteinAligner using this extended dataset. Experimental results (Figure S4) shows that the performance of the with AF data and without AF data models is statistically indistinguishable. For datasets used in downstream tasks, we followed the train–test split strategies specified in the original papers from which the datasets were obtained whenever applicable. In cases where no predefined split was available, we adopted a standard 80:20 random split. Below, we provide a detailed description of the data splitting procedures for each dataset. For type I anti-CRISPR activity detection, we used a dataset^49^ comprising 227 Acr–CRISPR-Cas system pairs, including 132 positive (inhibitory) and 95 negative (non-inhibitory) examples. Following the setup in,^49^ we applied a random 80:20 split. For minimum inhibitory concentration (MIC) prediction of antimicrobial peptides, the original dataset^60^ for Gram-negative AMPs included only a training set. We therefore performed an 80:20 random split, yielding 3,695 peptides for training and 924 for testing. For pathogenic missense variant prediction, we followed the predefined balanced 50:50 split from the original source,^46^ consisting of 100 mutations in the training set and 100 in the test set. Finally, for thermostability prediction, we used the same train–test split as in,^67^ which contains 936 proteins for training and 104 for testing. For peptide bioactivity prediction, we used the train–test splits provided by,^68^ who curated and partitioned the datasets for each specific bioactivity task. Specifically, for the dipeptidylpeptidase IV (DPP-IV) inhibitory peptide prediction task, the goal is to identify peptides that inhibit DPP-IV activity.^69^ We used the iDPPIV-SCM dataset,^69^ containing 532 inhibitory peptides and 532 non-inhibitory peptides for training, and 133 inhibitory peptides and 133 non-inhibitory peptides for testing. In the neuropeptide (NP) prediction task, the aim is to classify peptides as neuropeptides or non-neuropeptides.^52^ We used the PredNeuroP dataset,^52^ containing 1940 neuropeptides and 1940 non-neuropeptides for training, and 485 neuropeptides and 485 non-neuropeptides for testing. For the antiviral peptide prediction task, the objective is to predict whether a peptide has antiviral activity (preventative and therapeutic against viral infections).^53^ We utilized the ABPDiscover dataset,^70^ which includes 2321 antiviral peptides and 2321 non-antiviral peptides for training, and 623 antiviral peptides and 623 non-antiviral peptides for testing. In the antioxidant peptide prediction task, the aim is to classify peptides based on their antioxidant properties.^54^ We used the AnOxPePred dataset,^71^ containing 582 antioxidative peptides and 241 non-antioxidative peptides for training, with a test set comprising 28 antioxidative peptides and 61 non-antioxidative peptides. In the umami peptide prediction task, the goal is to determine whether a peptide elicits an umami taste.^55^ For this task, we used the iUmami-SCM dataset,^72^ with a training set of 112 umami peptides and 241 non-umami peptides, and a test set of 28 umami peptides and 61 non-umami peptides. In the blood–brain barrier peptide (BBP) prediction task, the objective is to classify whether a peptide can penetrate the blood–brain barrier (i.e., BBP).^56^ We employed the BBPpred dataset,^56^ consisting of 100 BBPs and 100 non-BBPs for training, and 19 BBPs and 19 non-BBPs for testing. The tumor T cell antigen prediction task aims to classify peptides capable of inducing a T cell immune response.^57^ We used the iTTCA-Hybrid dataset,^57^ including 470 antigenic peptides and 318 non-antigenic peptides for training, with 122 antigenic peptides and 75 non-antigenic peptides for testing. These datasets include a range of short peptides annotated with diverse functional labels.

#### Encoders in ProteinAligner

ProteinAligner utilizes ESM-2 (650M)^20^ to learn representations for protein sequences. ESM-2 (650M), a protein language model, was pretrained on 65 million protein sequences from UniRef50^73^ by predicting masked amino acids. The model features 33 transformer layers and an embedding dimension of 1280, allowing it to effectively capture the complexities inherent in protein sequences. To encode protein structures, ProteinAligner employs ESM-IF1,^9^ a model trained to address the inverse folding problem - predicting the amino acid sequence from a protein’s backbone atom coordinates. ESM-IF1 comprises an encoder and a decoder, where the encoder extracts a representation vector from the input structure, which is then fed into the decoder to generate the corresponding sequence. ProteinAligner utilizes only the encoder from ESM-IF1, omitting the decoder component. The encoder is composed of four Geometric Vector Perceptron Graph Neural Network (GVP-GNN)^41^ layers for geometric feature extraction, followed by eight transformer encoder layers to capture long-range interactions between these features. ESM-IF1 was trained on 12 million AlphaFold2^6^ computed protein structures and 16,000 experimentally verified structures, along with their associated sequences from the UniRef50 dataset.^73^ The text encoder is a transformer model comprising eight layers and a total of 78 million parameters. To aggregate per-position embeddings into a single representation for the entire protein sequence, we use the output embedding of the beginning-of-sequence (BOS) token from the ESM-2 encoder. The BOS token is prepended to each input sequence and attends to all positions during encoding, thereby serving as a global summary that captures information across the full sequence. For structure-based representations extracted by the ESM-IF1 encoder, we compute the mean of the per-residue embeddings across all positions, providing an overall summary of the 3D structural context. For functional text, following,^74^ we use the embedding of the end-of-sequence (EOS) token, which is appended to the end of the tokenized input, to summarize the textual information. Each modality-specific summary is then passed through a lightweight projection head consisting of a LayerNorm^75^ and a single-layer multi-layer perceptron, yielding a fixed-size representation vector. These vectors are used for contrastive learning across modalities.

#### ProteinAligner pretraining

Given a protein sequence *S* and a protein structure *R*, we employ the sequence encoder *E*_*s*_(·) and structure encoder *E*_*r*_(·) to extract representation vectors s = *E*_*s*_(*S*) and r = *E*_*r*_(*R*) for *S* and *R*, respectively. To ensure that the representations are similar when *S* and *R* belong to the same protein, and dissimilar when they do not, we minimize the InfoNCE^32^ contrastive learning loss:

(Equation 1) $$L_{s,r}=-\log\frac{\;\exp\;\left(s_{i}^{⊤}r_{i}/\tau \right)}{\;\exp\;\left(s_{i}^{⊤}r_{i}/\tau \right)+\sum_{j\neq i}\;\exp\;\left(s_{i}^{⊤}r_{j}/\tau \right)}\text{,}$$

where s_*i*_ and r_*i*_ represent the sequence and structure representations of the same protein *i*, while s_*i*_ and r_*j*_ represent the representations of different proteins *i* and *j*. This loss function encourages the alignment of s_*i*_ and r_*i*_ and discourages the similarity between s_*i*_ and r_*j*_. The temperature parameter *τ* controls the sharpness of the softmax distribution. Similarly, given a protein sequence *S* and a protein text description *T*, we use the sequence encoder *E*_*s*_(·) and the text encoder *E*_*t*_(·) to obtain representation vectors s = *E*_*s*_(*S*) and t = *E*_*t*_(*T*) for *S* and *T*, respectively. To ensure that the representations are similar when *S* and *T* correspond to the same protein, and dissimilar when they do not, we minimize the following InfoNCE contrastive learning loss:

(Equation 2) $$L_{s,t}=-\log\frac{\;\exp\;\left(s_{i}^{⊤}t_{i}/\tau \right)}{\;\exp\;\left(s_{i}^{⊤}t_{i}/\tau \right)+\sum_{j\neq i}\;\exp\;\left(s_{i}^{⊤}t_{j}/\tau \right)}\text{.}$$

For specific experimental settings, we minimized the sum of the two loss functions with equal weights. The temperature parameter *τ* was configured to 0.07. Pretraining was carried out over 20 epochs with a total batch size of 80 using 40 A100 GPUs. For distributed training, we employed Distributed Data Parallel (DDP)^76^ rather than Fully Sharded Data Parallel (FSDP),^77^ due to the increased inter-device communication overhead associated with FSDP. In our experiments, DDP offered a favorable trade-off between performance and scalability. On the other hand, our framework is fully compatible with FSDP and can be adapted to it if larger-scale protein encoders are used. During pretraining, each batch contains both sequence–structure and sequence–text pairs. For example, when the batch size is set to 80, we sample 80 sequence–structure pairs and 80 sequence–text pairs per iteration. The InfoNCE loss is then computed separately for each modality pair and averaged. The sampling of pairs is performed uniformly at random from the available paired data. We optimized the model weights using the AdamW optimizer,^78^ with an initial learning rate of 5 × 10^−6^, weight decay of 1 × 10^−4^, and betas of (0.9,0.95). The learning rate was dynamically adjusted throughout pretraining via the CosineAnnealingLR scheduler.^79^ The training dynamics of the contrastive learning losses for sequence–structure pairs, sequence–text pairs, and their combined loss can be found in Figure S6. Moreover, we also evaluate whether incorporating direct structure–text alignment (STA) improves performance (Figure S4), we extended the pretraining framework to include an additional STA objective alongside the existing structure–sequence and sequence–text alignments.

#### Downstream tasks

To evaluate the representational power of ProteinAligner, a diverse suite of downstream tasks was conducted across functional, biophysical, and clinical domains. The framework was tested on predicting pathogenic missense variants, estimating protein thermostability classes, and detecting type I anti-CRISPR activities. Additionally, the model’s performance was assessed on identifying potent bioactive peptides across seven distinct bioactivities and predicting the minimum inhibitory concentration (MIC) of antimicrobial peptides against E. coli. Finally, the model was evaluated on protein fitness prediction using the ProteinGym benchmark, as well as through zero-shot and homology-filtered settings to ensure robust generalization to novel proteins. And, the detailed architecture for all downstream tasks can be found in Figure S7.

#### Pathogenic missense variants prediction

The overall model architecture is depicted in Figure S7A. For this task, the sequence encoder pretrained by ProteinAligner was fine-tuned. The classification module was based on a multi-layer perceptron, which comprises a fully connected layer with a hidden state size of 1280, a dropout layer with a probability of 0.5, a leaky ReLU activation,^80^ and a second fully connected layer with a softmax activation function. We employed the Adam optimizer with a learning rate of 1 × 10^−4^ and a weight decay of 1 × 10^−3^, training the model for a maximum of 200 epochs with a batch size of 32. To mitigate overfitting, we applied an early stopping strategy: if validation performance did not improve over 10 consecutive epochs, training was halted, and the model checkpoint with the best validation performance was retained as the final model. The metrics used to evaluate model performance in this task include precision, recall rate, and F1 score.

#### Thermostability prediction

The overall model architecture is depicted in Figure S7B. The structure encoder, pretrained using ProteinAligner, was fine-tuned for this task. The classification module, implemented as a multi-layer perceptron (MLP), includes a fully connected layer with a hidden dimension of 128, followed by layer normalization,^81^ a ReLU activation, and a second fully connected layer to produce the classification logits.

#### Type I anti-CRISPR activity detection

The overall model architecture for this task is illustrated in Figure S7C. The sequence encoder pretrained by ProteinAligner was fine-tuned using data specific to this task. The classification module was based on a CNN, which was composed of two 1D convolutional layers, each with a stride of 1 and a kernel size of 7. The first convolutional layer takes an input of 1280 channels and outputs 4 channels. The second convolutional layer maintains the same input and output dimensions. Batch normalization^82^ and ReLU activation^83^ are applied after each convolutional layer. Following the convolutional layers, two fully connected layers, each with a hidden size of 4, are employed for final classification. During training, we optimized model weights using the Adam^84^ optimizer with an initial learning rate of 3 × 10^−3^, over a maximum of 250 epochs with a batch size of 32. To prevent overfitting, we employed early stopping when the decrease in training loss fell below 0.005 and applied weight decay, starting at 0.01 and gradually reducing to 0.001. We also performed a hyperparameter sweep on the dropout rate,^85^ exploring values between 0.3 and 0.5. Additionally, we implemented a learning rate reduction strategy, decreasing the rate by a factor of 0.9 if validation performance did not improve for 10 consecutive epochs. The model’s performance was evaluated using accuracy and F1 score.

#### Identification of potent bioactive peptides

The overall model architecture is illustrated in Figure S7D. The sequence encoder, pretrained by ProteinAligner, was fine-tuned for each of the eight tasks. The classification module employs a convolutional neural network (CNN) with six layers, structured as follows: a 1D convolutional layer (kernel size = 3, stride = 1, padding = 2), followed by BatchNorm and ReLU activation; a max pooling layer (kernel size = 2, padding = 1) and a dropout layer with a probability of 0.15; another 1D convolutional layer (kernel size = 3, stride = 1, padding = 2), followed by BatchNorm and ReLU activation; a max pooling layer (kernel size = 2, padding = 1) and a dropout layer with a probability of 0.15; a fully connected layer with a hidden state size of 64, followed by ReLU activation and a dropout layer (probability = 0.15); and finally, a fully connected binary classification layer with sigmoid activation. During training, we optimized the model using stochastic gradient descent (SGD) with a learning rate of 1 × 10^−2^, momentum of 0.5, and no weight decay, over 200 epochs. Additionally, we applied step decay for learning rate adjustment and utilized early stopping based on validation accuracy, halting training if no improvement was observed for 40 consecutive epochs. Model performance was assessed using several metrics, including accuracy (ACC), balanced accuracy (BACC),^58^ sensitivity (SN), specificity (SP), Matthews correlation coefficient (MCC),^59^ and area under the ROC curve (AUC).

#### Minimum inhibitory concentration (MIC) value prediction

The model architecture is illustrated in Figure S7E. The sequence encoder, pretrained with ProteinAligner, was fine-tuned to address this task. The classification module is a multi-layer perceptron (MLP) consisting of two fully connected layers, with a hidden size of 256 and a ReLU activation function. We employed the Adam optimizer with an initial learning rate of 1 × 10^−4^, training the model for 200 epochs. Throughout the training process, the learning rate was dynamically adjusted at each epoch using the LambdaLR^86^ scheduler. To assess the model’s performance, we used mean squared error (MSE) as the evaluation metric.

#### Supervised protein fitness prediction

We evaluated ProteinAligner and baseline models on the ProteinGym Deep Mutational Scanning (DMS) benchmark, which comprises 217 assays with quantitative fitness measurements.^87^ In the supervised setting, each model was trained on a subset of variants from each assay and evaluated on held-out single-mutation variants from the same assay. Predictive performance on unseen variants was assessed using Spearman’s rank correlation coefficient (Spearman), Pearson correlation coefficient (Pearson), and coefficient of determination (*R*^2^). To reduce computational cost, we randomly selected 15 single-substitution assays (listed in Table S1). The model architecture used for this task is shown in Figure S7F: the sequence embedding produced by ProteinAligner is passed through a one-dimensional convolutional layer (kernel size 7, stride 1, same padding), followed by dropout (rate 0.1), a ReLU activation, and a single-layer multilayer perceptron. Fine-tuning was performed using the AdamW optimizer (learning rate 3 × 10^−4^, weight decay 5 × 10^−2^, batch size 64) with a cosine annealing schedule and 100 warm-up steps, for a total of 10,000 training steps. Importantly, we assess the influence of prediction heads on downstream task performance, results (Figure S5) shows that ProteinAligner consistently outperformed baseline models, indicating that its performance gains are robust to the choice of prediction head.

### Quantification and statistical analysis

All statistical analyses were performed in Python using *scipy.stats*. Unless otherwise noted, each experiment was repeated for *n* = 5 independent runs per task with different random seeds (no cross-validation). We report the mean performance across runs, and error bars indicate the standard deviation (SD) over the five runs. Statistical significance between our method and the baseline was assessed using a two-sided paired *t* test on per-seed task metrics (paired by seed). We defined statistical significance as *p* < 0.05 without multiple-comparison correction. We used official dataset splits when provided; otherwise, we created a random split with a fixed seed and applied the same split to all compared methods. No runs were excluded from analysis.

## Supplemental information

Document S1. Figures S1–S7 and Table S1

Document S2. Article plus supplemental information
