# Supplementary material for: A tri-modal contrastive learning framework for protein representation learning
Source: Cell Rep Methods. 2026 Apr 15;6(5):101407. doi: 10.1016/j.crmeth.2026.101407 (PMC13198128; doi:10.1016/j.crmeth.2026.101407)
Supplement: Document S1. Figures S1–S7 and Table S1 [file mmc1.pdf]

Cell Reports Methods, Volume *6*

Supplemental information

A tri-modal contrastive learning framework for protein representation learning

Li Zhang, Han Guo, Leah Schaffer, Young Su Ko, Digvijay Singh, Hamid Rahmani, Danielle Grotjahn, Elizabeth Villa, Michael Gilson, Wei Wang, Trey Ideker, Eric Xing, and Pengtao Xie

Table S1. **Data distribution for protein fitness prediction.** List of assays randomly selected from the ProteinGym benchmark (15 out of 217 total assays). Related to STAR Methods.

Data source	Num of observed fitness
A0A140D2T1_ZIKV_Sourisseau_2019	9576
A0A247D711_LISMN_Stadelmann_2021	1653
A4D664_9INFA_Soh_2019	14421
CAS9_STRP1_Spencer_2017_positive	8117
D7PM05_CLYGR_Somermeyer_2022	1169
ENV_HV1B9_DuenasDecamp_2016	375
GLPA_HUMAN_Elazar_2016	245
KCNE1_HUMAN_Muhammad_2023_function	2315
ODP2_GEOSE_Tsuboyama_2023_1W4G	669
PKN1_HUMAN_Tsuboyama_2023_1URF	1301
Q59976_STRSQ_Romero_2015	2999
REV_HV1H2_Fernandes_2016	2147
SBI_STAAM_Tsuboyama_2023_2JVG	1025
TNKS2_HUMAN_Tsuboyama_2023_5JRT	1118
TPK1_HUMAN_Weile_2017	3181
YNZC_BACSU_Tsuboyama_2023_2JVD	714

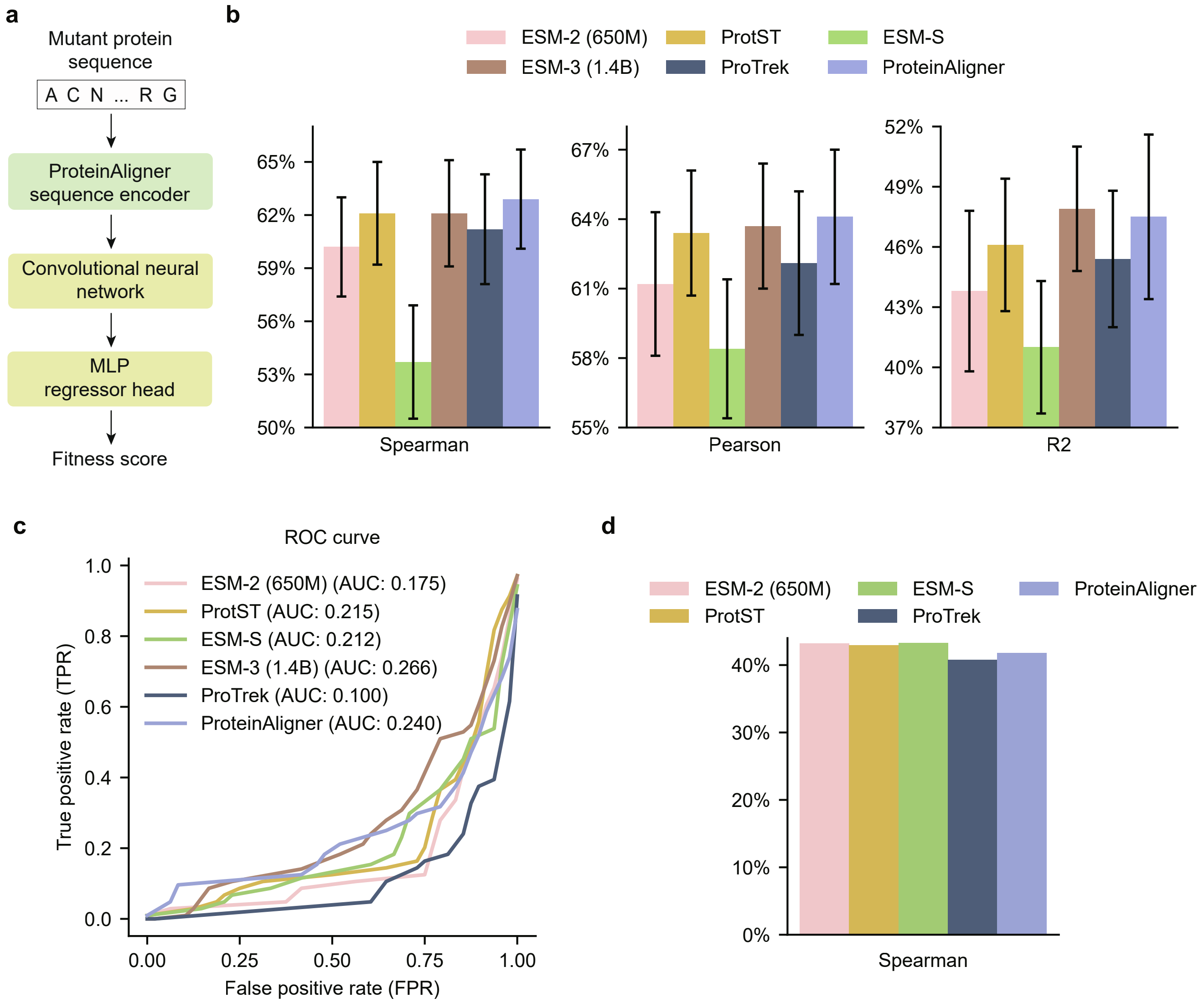

Figure S1. **ProteinAligner demonstrates competitive performance in protein fitness prediction.** **a**, Fine-tuning architecture for using the pretrained ProteinAligner sequence encoder to predict protein fitness. Experiments were conducted on a subset of the ProteinGym benchmark[1], consisting of 15 randomly selected single-substitution DMS assays from a total of 217. The selected assays are listed in Table S1, with an average of 3,402 fitness measurements per assay. Each assay was evaluated using Spearman and Pearson correlation coefficients, and coefficient of determination, with higher values indicating better performance. **b**, ProteinAligner outperformed all baselines across all evaluation metrics with the exception of a slight deficit to ESM-3 in R^2. Data are represented as "mean $\pm$ standard deviation" over three independent runs. **ProteinAligner achieves competitive performance in zero-shot prediction settings.** **c**, Following Meier et al.[2], we performed zero-shot pathogenicity prediction by encoding wild-type and mutant sequences to extract vectors at mutation sites. Using the ESM-2 head, we calculated the log-probability difference between residues as a pathogenicity score, classifying mutations based on a fixed threshold. ProteinAligner outperformed all baseline methods in zero-shot pathogenic missense variant prediction, as measured by area under the ROC curve. **d**, Following Jiang et al.[3], we performed zero-shot thermostability prediction using 66 single-site mutation assays from MPTherm[4], FireProtDB[5], and ProThermDB[6]. Using the ESM-2 prediction head, we calculated the log-probability difference between mutant and wild-type residues as a proxy for melting temperature impact. Performance was evaluated via Spearman's rank correlation against experimental data. ProteinAligner achieved Spearman correlation scores comparable to those of baseline methods in zero-shot protein thermostability prediction. Related to STAR Methods.

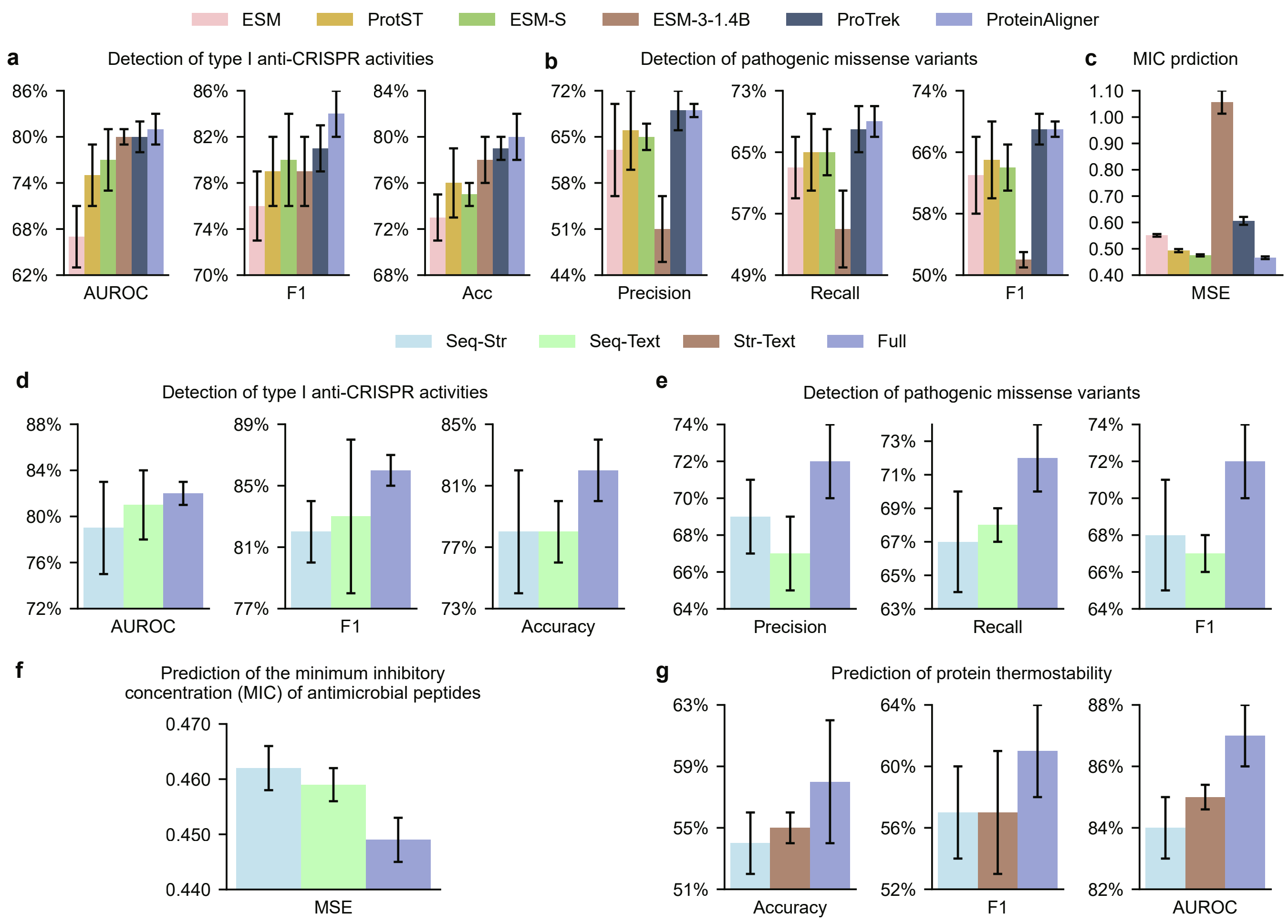

Figure S2. **Evaluation of ProteinAligner on homology-filtered test sets.** To assess ProteinAligner's generalization, we created new test sets using stringent homology filtering. Using BLASTp[7], we retained only proteins with less than 10% sequence identity (pident) to any pretraining sequences. This conservative threshold removes family-level and remote homology to ensure a robust evaluation. Results are shown for three representative downstream tasks: (a) detection of type I anti-CRISPR activity, (b) detection of pathogenic missense variants, and (c) prediction of antimicrobial peptide minimum inhibitory concentration (MIC). ProteinAligner consistently outperforms all baselines across these new test sets, demonstrating robust generalization to proteins with minimal sequence similarity to the pretraining data. Data are represented as "mean ± standard deviation" over three independent runs. Related to STAR Methods. **Ablation study demonstrating the contribution of each modality in ProteinAligner.** To evaluate each modality's contribution, we compared our tri-modal configuration against three bi-modal variants: Seq–Str, Seq–Text, and Str–Text, using identical training conditions. Structure encoders (from Seq–Str, Str–Text, and Full settings) were fine-tuned on thermostability prediction. All models were pretrained under identical conditions. **d–f**, Fine-tuning results of the sequence encoders on three downstream tasks: **(d)** pathogenic missense variant prediction, **(e)** type I anti-CRISPR activity detection, and **(f)** minimum inhibitory concentration (MIC) prediction of antimicrobial peptides. **g**, Fine-tuning results of the structure encoders on protein thermostability prediction. Across all tasks, the Full tri-modal configuration consistently outperforms the bi-modal variants. Data are represented as "mean ± standard deviation" over three independent runs. Related to Figure 4.

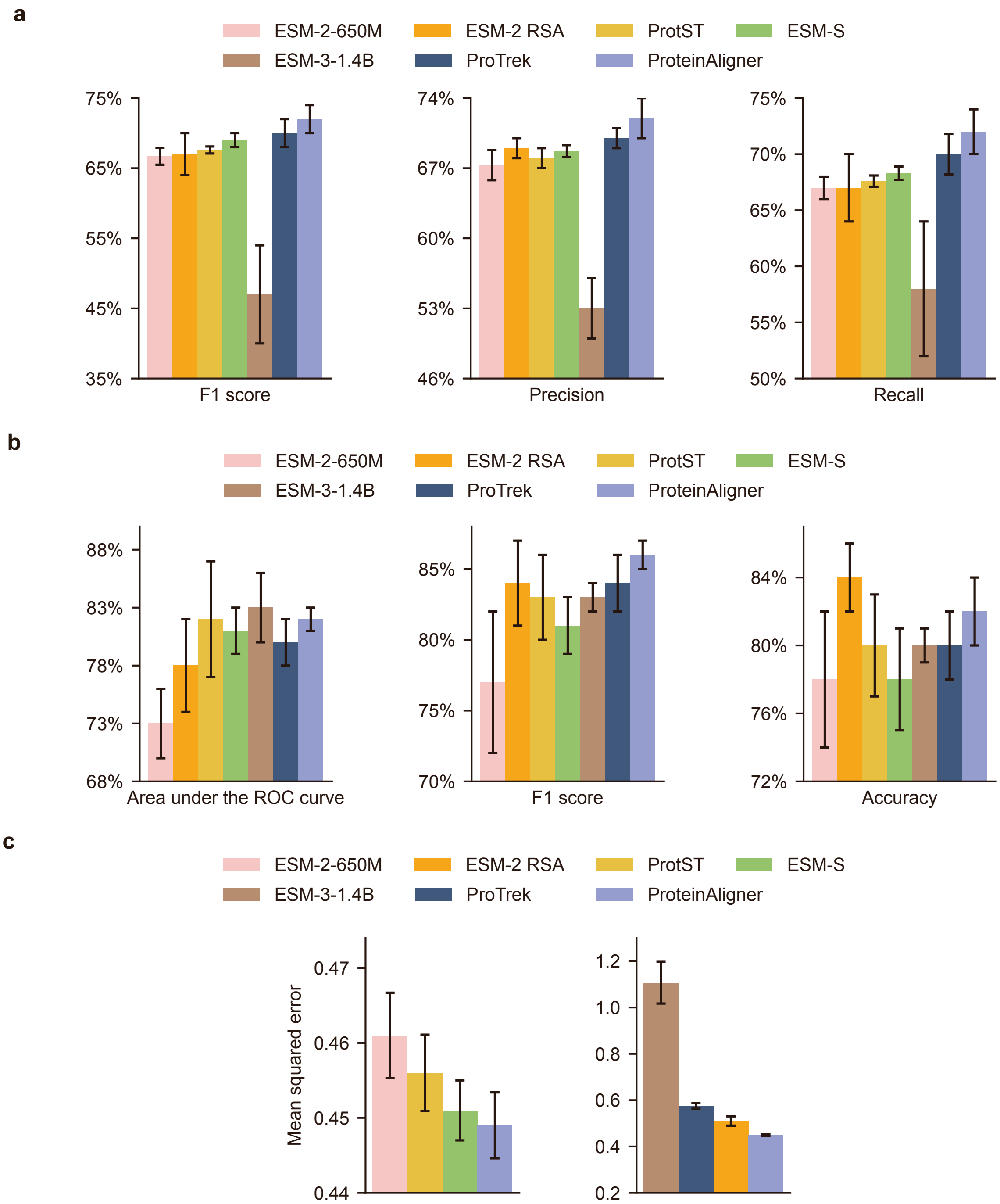

Figure S3. **Comparison between retrieval-based sequence augmentation and multimodal pretraining.** Gemini said To evaluate retrieval-based sequence augmentation versus multimodal integration, we compared ProteinAligner with the RSA approach[8] using ESM-2 (650M) as the backbone. For pathogenicity, anti-CRISPR, and MIC prediction tasks, we used BLASTp to retrieve the top five training sequences for each query. These were concatenated with the query, encoded via ESM-2, and the resulting vectors were averaged to form the final embedding for the task-specific prediction head. Performance comparison across three sequence-based downstream tasks: **(a)** pathogenic missense variant prediction, **(b)** type I anti-CRISPR activity detection, and **(c)** antimicrobial peptide minimum inhibitory concentration prediction. RSA underperforms compared to ProteinAligner in most metrics, except for type I anti-CRISPR detection. While RSA's sequence retrieval captures evolutionary similarity, it lacks the 3D biophysical context—such as steric effects and side-chain packing—essential for protein function. Data are represented as "mean ± standard deviation" over three independent runs. Related to Figure 2.

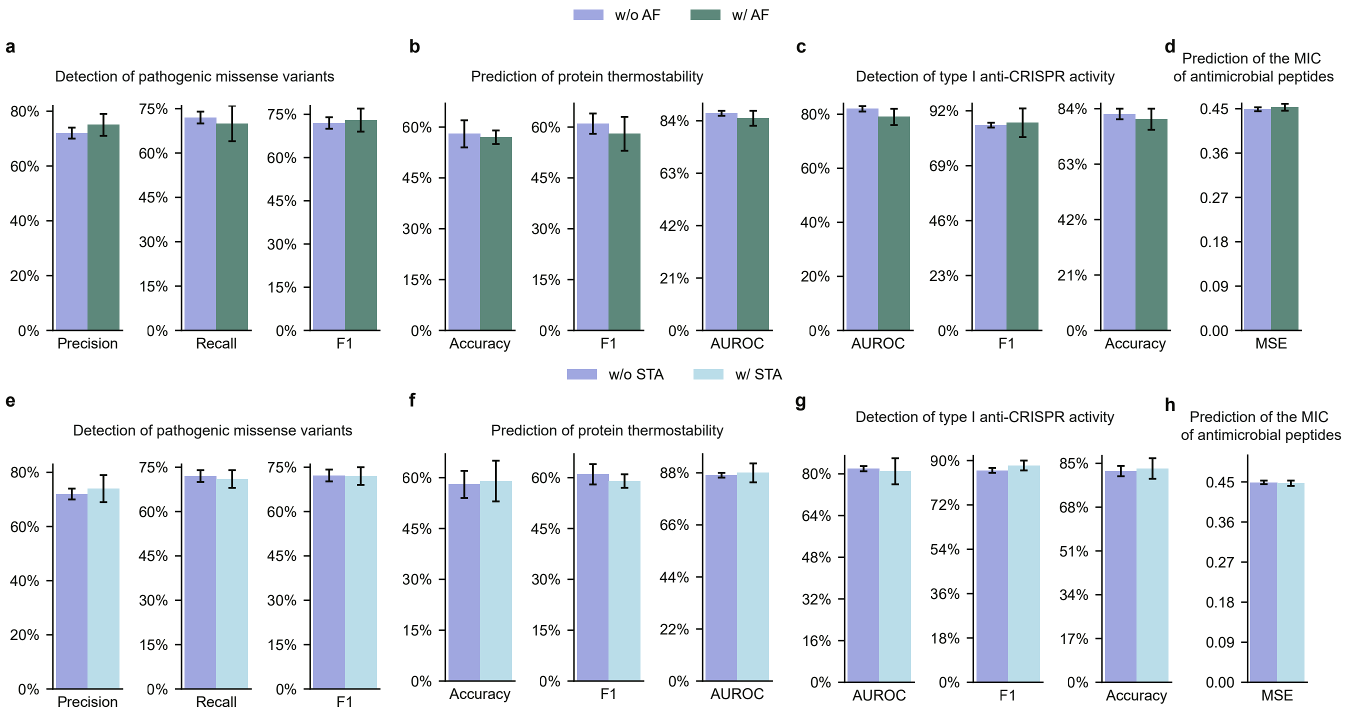

Figure S4. **Comparison between pretraining with and without structure–text alignment.** Performance comparison between the original ProteinAligner framework without direct structure–text alignment (w/o STA) and a modified version incorporating explicit structure–text alignment (w/ STA). Encoders pretrained under both settings were fine-tuned on four representative downstream tasks: **(a)** pathogenic missense variant prediction, **(b)** protein thermostability prediction, **(c)** type I anti-CRISPR activity detection, and **(d)** minimum inhibitory concentration (MIC) prediction of antimicrobial peptides. Performance for w/ STA and w/o STA is comparable, with no significant improvement from direct structure–text alignment. However, w/ STA increases computational cost by 27%. ProteinAligner achieves effective integration via the shared sequence anchor, which implicitly couples structure and text. Because the sequence acts as a sufficient intermediary, the explicit STA objective is redundant. The original sequence-anchored design is therefore more efficient without sacrificing accuracy. **Evaluating the effect of incorporating AlphaFold-predicted structures in pretraining.** To evaluate the impact of predicted structures, we expanded the pretraining corpus with 119,485 AlphaFold DB sequence–structure pairs (w/ AF data) and compared it to the original dataset (w/o AF data). Both models were trained under identical conditions and fine-tuned on four representative downstream tasks: **(e)** pathogenic missense variant prediction, **(f)** protein thermostability prediction, **(g)** type I anti-CRISPR activity detection, and **(h)** minimum inhibitory concentration (MIC) prediction of antimicrobial peptides. Performance for w/ AF data and w/o AF data is statistically identical, but adding AlphaFold data increases costs by 39%. This lack of gain is due to low informational diversity, as predicted structures are sequence-derived and thus redundant. Furthermore, local inaccuracies in flexible regions or side-chains in predicted models introduce noise, weakening cross-modal alignment compared to experimental structures. Data are represented as "mean $\pm$ standard deviation" over three independent runs. Related to Figure 2.

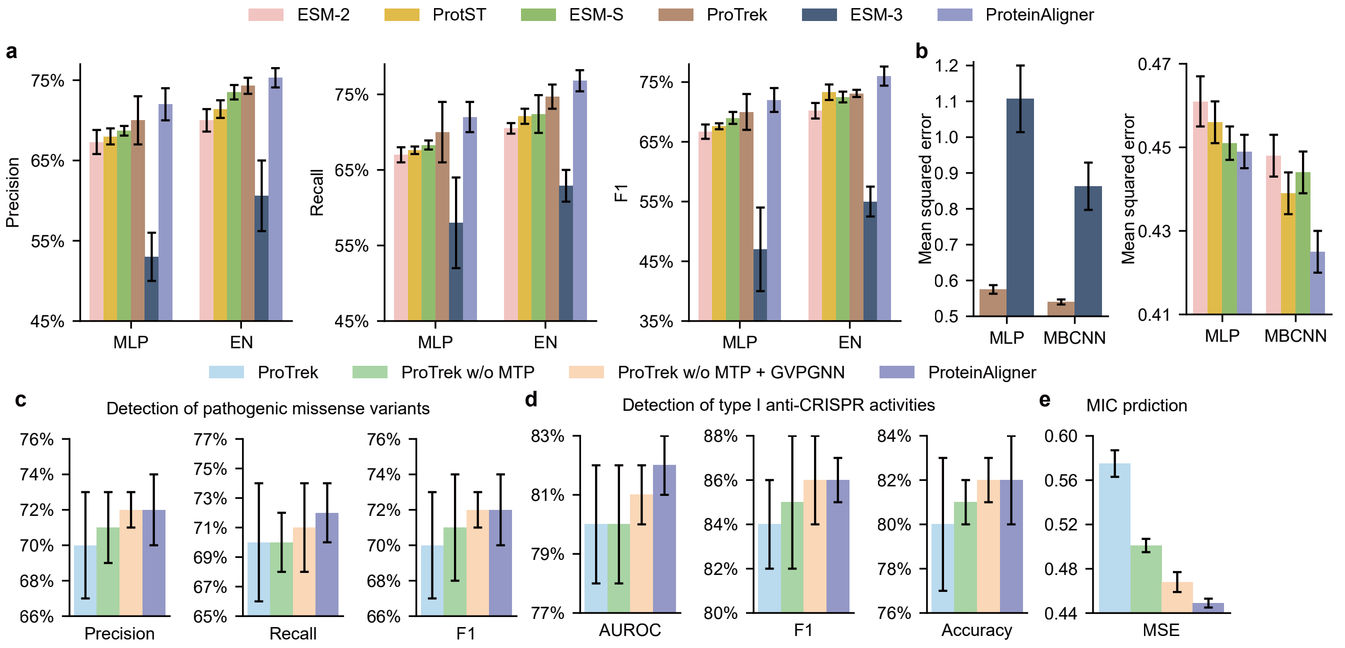

Figure S5. **ProteinAligner demonstrates robust performance across different prediction head architectures.** To assess how prediction heads influence performance, we tested classification and regression on two tasks. For pathogenicity prediction (classification), we compared a Multi-Layer Perceptron against a linear Elastic Net model[9] using PCA-reduced embeddings, following[10]. For MIC regression, we compared an MLP head with a multi-branch CNN architecture[11,12] optimized for peptide prediction. **a**, ProteinAligner achieved higher precision, recall, and F1 score than baseline models in pathogenic missense variant prediction when using either a multi-layer perceptron or an Elastic Net classifier as the prediction head. **b**, ProteinAligner yielded lower prediction errors than baselines in minimum inhibitory concentration (MIC) regression for antimicrobial peptides when using either a multi-layer perceptron (MLP) or a multi-branch convolutional neural network as the prediction head. The results indicate that its performance gains are robust to the choice of prediction head. While the architecture of the prediction head can affect the absolute level of performance, the improvements provided by ProteinAligner persisted regardless of the prediction head used. **Evaluating the impact of masked token prediction and continuous structural encoding on ProTrek performance.** To investigate prior multimodal framework limitations, we compared ProteinAligner with a modified ProTrek design. We removed the masked token prediction (MTP) objective from ProTrek (ProTrek w/o MTP). Performance comparison among (i) the original ProTrek model, (ii) ProTrek without masked token prediction (ProTrek w/o MTP), and (iii) ProTrek with both masked token prediction removed and the GVP-GNN structure encoder from ProteinAligner (ProTrek w/o MTP + GVPGNN). Removing the masked token prediction objective improves ProTrek performance across four downstream tasks: pathogenic missense variant prediction, protein thermostability prediction, type I anti-CRISPR activity detection, and antimicrobial peptide MIC prediction, indicating that multitask optimization conflicts hinder the original model. Replacing ProTrek's discrete structure encoder with a continuous GVP-GNN further improves performance, approaching that of ProteinAligner. These results support that contrastive-only optimization and continuous structural representations are key design factors underlying ProteinAligner's superior performance. Data are represented as "mean $\pm$ standard deviation" over three independent runs. Related to Figure 2.

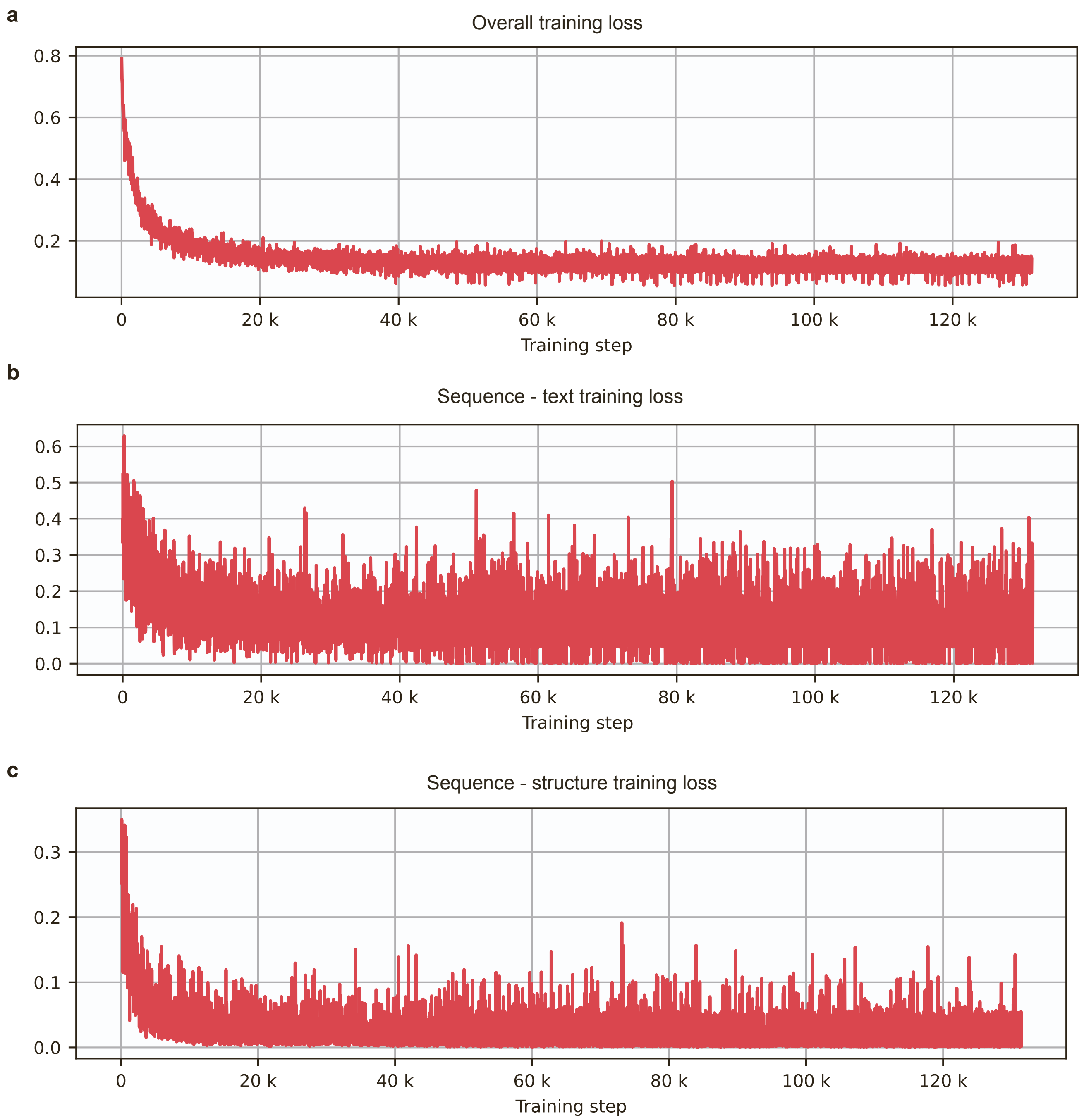

Figure S6. **Training dynamics of ProteinAligner.** **a**, Overall loss curve during pretraining. **b**, Sequence-text loss curve during pretraining. **c**, Sequence-structure loss curve during pretraining. The combined loss decreases smoothly and stabilizes around 0.1, indicating stable convergence. The sequence–structure loss shows a rapid and consistent decline, reaching approximately 0.05 within the first 20,000 training steps. The sequence–text loss exhibits moderate fluctuations in the early stages, which gradually diminish and stabilize near 0.15. Related to STAR Methods.

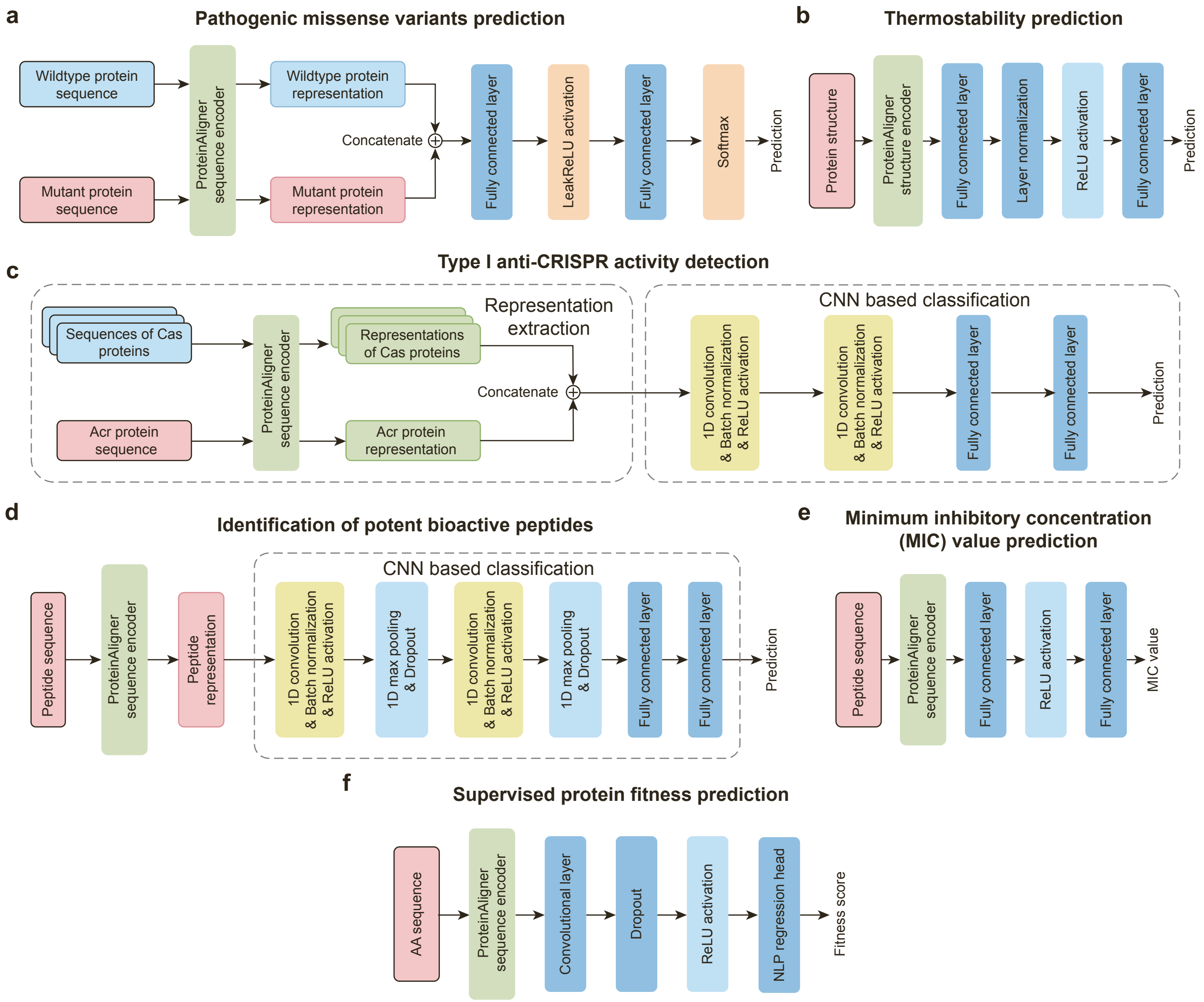

Figure S7. **Model architectures used in downstream tasks.** **a**, Model architecture used in pathogenic missense variants prediction. **b**, Model architecture used in thermostability prediction. **c**, Model architecture used in type I anti-CRISPR activity detection. **d**, Model architecture used for identifying potent bioactive peptides. **e**, Model architecture used for predicting minimum inhibitory concentration values. **f**, Model architecture used for supervised protein fitness prediction. Related to STAR Methods.

References

S1. Notin, P., Kollasch, A., Ritter, D., Van Niekerk, L., Paul, S., Spinner, H., Rollins, N., Shaw, A., Orenbuch, R., Weitzman, R. et al. (2023). Proteingym: Large-scale benchmarks for protein fitness prediction and design. Advances in Neural Information Processing Systems *36*, 64331–64379.

S2. Meier, J., Rao, R., Verkuil, R., Liu, J., Sercu, T., and Rives, A. (2021). Language models enable zero-shot prediction of the effects of mutations on protein function. Advances in neural information processing systems *34*, 29287–29303.

S3. Jiang, F., Li, M., Dong, J., Yu, Y., Sun, X., Wu, B., Huang, J., Kang, L., Pei, Y., Zhang, L. et al. (2024). A general temperature-guided language model to design proteins of enhanced stability and activity. Science Advances *10*, eadr2641.

S4. Kulandaisamy, A., Sakthivel, R., and Gromiha, M.M. (2021). Mptherm: database for membrane protein thermodynamics for understanding folding and stability. Briefings in Bioinformatics *22*, 2119–2125.

S5. Stourac, J., Dubrava, J., Musil, M., Horackova, J., Damborsky, J., Mazurenko, S., and Bednar, D. (2021). Fireprotdb: database of manually curated protein stability data. Nucleic acids research *49*, D319–D324.

S6. Nikam, R., Kulandaisamy, A., Harini, K., Sharma, D., and Gromiha, M.M. (2021). Prothermdb: thermodynamic database for proteins and mutants revisited after 15 years. Nucleic acids research *49*, D420–D424.

S7. Camacho, C., Coulouris, G., Avagyan, V., Ma, N., Papadopoulos, J., Bealer, K., and Madden, T.L. (2009). Blast+: architecture and applications. BMC bioinformatics *10*, 421.

S8. Ma, C., Zhao, H., Zheng, L., Xin, J., Li, Q., Wu, L., Deng, Z., Lu, Y., Liu, Q., and Kong, L. (2023). Retrieved sequence augmentation for protein representation learning. bioRxiv pp. 2023–02.

S9. Zou, H., and Hastie, T. (2005). Regularization and variable selection via the elastic net. Journal of the Royal Statistical Society Series B: Statistical Methodology *67*, 301–320.

S10. Fan, X., Pan, H., Tian, A., Chung, W.K., and Shen, Y. (2023). Shine: protein language model-based pathogenicity prediction for short inframe insertion and deletion variants. Briefings in Bioinformatics *24*, bbac584.

S11. Yan, J., Zhang, B., Zhou, M., Campbell-Valois, F.X., and Siu, S.W. (2023). A deep learning method for predicting the minimum inhibitory concentration of antimicrobial peptides against escherichia coli using multi-branch-cnn and attention. Msystems *8*, e00345–23.

S12. Yan, J., Zhang, B., Zhou, M., Kwok, H.F., and Siu, S.W. (2022). Multi-branch-cnn: Classification of ion channel interacting peptides using multi-branch convolutional neural network. Computers in Biology and Medicine *147*, 105717.
